# TRPA1 promotes the maturation of embryonic stem cell-derived cardiomyocytes by regulating mitochondrial biogenesis and dynamics

**DOI:** 10.1186/s13287-023-03388-3

**Published:** 2023-06-07

**Authors:** Qianqian Ding, Xianji Liu, Yanxiang Qi, Xiaoqiang Yao, Suk Ying Tsang

**Affiliations:** 1 School of Life Sciences, The Chinese University of Hong Kong, Shatin, Hong Kong SAR China; 2 School of Biomedical Sciences, The Chinese University of Hong Kong, Shatin, Hong Kong SAR China; 3 State Key Laboratory of Agrobiotechnology, The Chinese University of Hong Kong, Shatin, Hong Kong SAR China; 4 Key Laboratory for Regenerative Medicine, Ministry of Education, The Chinese University of Hong Kong, Shatin, Hong Kong SAR China; 5 Institute for Tissue Engineering and Regenerative Medicine, The Chinese University of Hong Kong, Shatin, Hong Kong SAR China

**Keywords:** Embryonic stem cell-derived cardiomyocytes, Cardiomyocyte maturation, Transient receptor potential ankyrin 1 channel, Mitochondria, Peroxisome proliferator-activated receptor gamma coactivator-1α

## Abstract

**Background:**

Cardiomyocytes derived from pluripotent stem cells (PSC-CMs) have been widely accepted as a promising cell source for cardiac drug screening and heart regeneration therapies. However, unlike adult cardiomyocytes, the underdeveloped structure, the immature electrophysiological properties and metabolic phenotype of PSC-CMs limit their application. This project aimed to study the role of the transient receptor potential ankyrin 1 (TRPA1) channel in regulating the maturation of embryonic stem cell-derived cardiomyocytes (ESC-CMs).

**Methods:**

The activity and expression of TRPA1 in ESC-CMs were modulated by pharmacological or molecular approaches. Knockdown or overexpression of genes was done by infection of cells with adenoviral vectors carrying the gene of interest as a gene delivery tool. Immunostaining followed by confocal microscopy was used to reveal cellular structure such as sarcomere. Staining of mitochondria was performed by MitoTracker staining followed by confocal microscopy. Calcium imaging was performed by fluo-4 staining followed by confocal microscopy. Electrophysiological measurement was performed by whole-cell patch clamping. Gene expression was measured at mRNA level by qPCR and at protein level by Western blot. Oxygen consumption rates were measured by a Seahorse Analyzer.

**Results:**

TRPA1 was found to positively regulate the maturation of CMs. TRPA1 knockdown caused nascent cell structure, impaired Ca^2+^ handling and electrophysiological properties, and reduced metabolic capacity in ESC-CMs. The immaturity of ESC-CMs induced by TRPA1 knockdown was accompanied by reduced mitochondrial biogenesis and fusion. Mechanistically, we found that peroxisome proliferator-activated receptor gamma coactivator-1α (PGC-1α), the key transcriptional coactivator related to mitochondrial biogenesis and metabolism, was downregulated by TRPA1 knockdown. Interestingly, overexpression of PGC-1α ameliorated the halted maturation induced by TRPA1 knockdown. Notably, phosphorylated p38 MAPK was upregulated, while MAPK phosphatase-1 (MKP-1), a calcium-sensitive MAPK inhibitor, was downregulated in TRPA1 knockdown cells, suggesting that TRPA1 may regulate the maturation of ESC-CMs through MKP-1-p38 MAPK-PGC-1α pathway.

**Conclusions:**

Taken together, our study reveals the novel function of TRPA1 in promoting the maturation of CMs. As multiple stimuli have been known to activate TRPA1, and TRPA1-specific activators are also available, this study provides a novel and straightforward strategy for improving the maturation of PSC-CMs by activating TRPA1. Since a major limitation for the successful application of PSC-CMs for research and medicine lies in their immature phenotypes, the present study takes a big step closer to the practical use of PSC-CMs.

**Graphical abstract:**

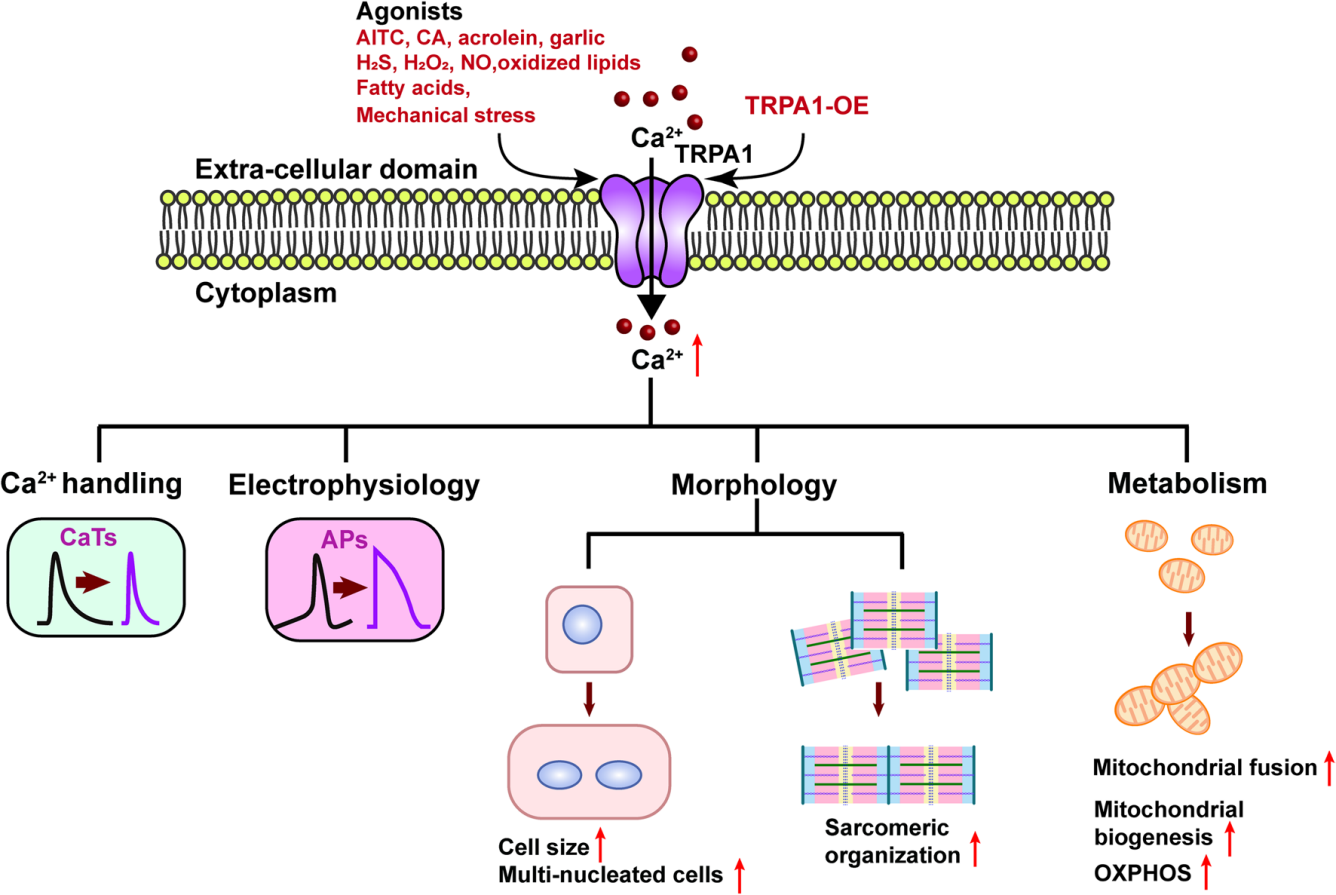

**Supplementary Information:**

The online version contains supplementary material available at 10.1186/s13287-023-03388-3.

## Introduction

Cardiovascular diseases are one of the leading causes of morbidity and mortality globally. Many forms of cardiovascular diseases result in the progressive loss of myocytes by apoptosis or necrosis, which ultimately progress to heart failure and death. Since the heart shows limited regenerative capacity, it is vital to seek new approaches to augment the regenerative capacity of the injured heart. Cardiomyocytes (CMs) derived from pluripotent stem cells (PSC-CMs) exhibit similar molecular, electrical, mechanical, and ultrastructural features as adult CMs and can be generated routinely with high yield; PSC-CMs have therefore hold great promise to be used in drug screening and heart regeneration therapies [1, 2]. However, a limitation for the successful application of PSC-CMs for research and medicine lies in their fetal-like phenotype [3]. Compared with adult CMs, PSC-CMs exhibit small cell size with less developed sarcomeres, reduced proportion of binucleated or multi-nucleated cells, immature Ca^2+^ kinetics and electrophysiological properties, and less mitochondrial volume and more reliance on glycolysis instead of oxidative phosphorylation for ATP production [4]. Thus, the next challenge in the field is to develop strategies to improve the maturation of PSC-CMs.

Multiple approaches have been proposed to enhance the maturation of PSC-CMs, including long-term culturing, 3-dimensional tissue engineering, and mechanical loading [5]. Recent studies have revealed that supplementing PSC-CMs with fatty acids, high oxidative substrate, or hormones can also improve their maturation [6–8]. Although the underlying mechanisms and participating factors involved in those processes have not been well-explored, these interventions recapitulate aspects of metabolic transitions during cardiac development. The energy supply of undifferentiated PSCs mainly depends on anaerobic glycolysis, which has to be transformed into a more efficient mitochondrial oxidative metabolism to secure cardiac specification and excitation–contraction coupling (ECC) during maturation [9]. In particular, the mitochondria also undergo structural maturation, transitioning from small, fragmented organelles to large networks with developed cristae capable of high oxidative capacity needed to produce enough ATP to meet the increased energy demand [10]. A complex system comprised of regulatory factors and energy metabolic machinery is required for the coordinated control of cardiac mitochondrial biogenesis, maturation, and function [11]. Peroxisome proliferator-activated receptor gamma coactivator-1α (PGC-1α) is the key transcriptional coactivator regulating mitochondrial biogenesis and metabolism. Liu et al. have demonstrated that the PGC-1α activator induced more mature energy metabolism, increased sarcomere length, improved calcium handling, and enhanced intercellular connectivity of CMs [12]. Murphy et al. have shown that PGC-1α drove CM maturation through YAP1 and SF3B2 [13].

Transient receptor potential ankyrin 1 (TRPA1), a member of the transient receptor potential (TRP) channel family, is a Ca^2+^-permeable non-selective cation channel. A remarkable feature of TRPA1 is its long N-terminal region with 14–18 predicted ankyrin repeats followed by a six-transmembrane domain [14]. These ankyrin repeats are essential for protein–protein interactions and insertion of the channel into the plasma membrane, as well as sensing both environmental and endogenous stimuli, including pungent natural compounds, environmental irritants, oxidative stress, mechanical stress, and fatty acids [15–17]. TRPA1 integrates multiple stimuli and transduces the stimuli to activate downstream signaling pathways via Ca^2+^ entry. Similar to most of the TRP channels, TRPA1 has mainly been described to express and function in the nervous system. Emerging evidence has suggested that TRPA1 plays an essential role in the development and progression of several cardiovascular conditions, including atherosclerosis, heart failure, myocardial ischemia–reperfusion injury, myocardial fibrosis, arrhythmia, vasodilation, and hypertension [18]. However, the role of TRPA1 in cardiac maturation has not been studied. We hypothesized that TRPA1 regulates the maturation of cardiomyocytes. In addition, based on the fact that TRPA1 mediates Ca^2+^ homeostasis and redox signaling, we hypothesized that the function of TRPA1 is linked to mitochondria. In this current study, we revealed that TRPA1 promotes CM maturation through regulating mitochondrial biogenesis and dynamics. These novel findings are important for us to understand the process of cardiac maturation and provide us with new insights into the strategy for enhancing the maturation of PSC-CMs.

## Methods

An expanded Methods section is available in Additional file 1.

### mESC culture and cardiac differentiation

mESC line D3 (ATCC, Manassas, VA, USA) was maintained in an undifferentiated state on irradiated mouse embryonic fibroblasts (MEFs) as previously described [19–21] where isolation of MEFs was approved by the Animal Experimentation Ethics Committee, the Chinese University of Hong Kong and conformed to Guide for the Care and Use of Laboratory Animals published by the United States National Institutes of Health (NIH Publication No. 80-23, revised 2011). The cells were cultured in an undifferentiation medium, which contained DMEM supplemented with 15% heat-inactivated FBS (HyClone, GE Healthcare, South Logan, UT, USA), 0.1 mM β-mercaptoethanol (Sigma-Aldrich, Darmstadt, Germany), 2 mM L-glutamine, 0.1 mM non-essential amino acids, 1% v/v penicillin–streptomycin and 1000 U/mL leukemia inhibitory factor (Chemicon, Millipore, Billerica, MA, USA). Differentiation of mESCs into CMs was performed by the hanging drop method as previously described [19–21].

### Isolation of mESC-CMs

On day (7 + 4), mESC-CMs were isolated as previously described [19–21]. The isolated mESC-CMs were plated on cover glass (Thermo Fisher Scientific) in a 24-well plate or confocal dishes (MatTek, Ashland, MA, USA) that have been pre-coated with 20 μg/mL laminin (Thermo Fisher Scientific) in 0.1% gelatin in 37 °C, 5% CO_2_ incubator.

TRPA1 activator or blocker treatment experiments were performed on day (7 + 5). For experiments involving TRPA1 knockdown or TRPA1 knockdown concomitant with PGC-1α overexpression, mESC-CMs were infected with adenoviruses on day (7 + 5) and the experiments were performed on day (7 + 9). For TRPA1 or PGC-1α overexpression experiments, mESC-CMs were infected with adenoviruses on day (7 + 5) and the experiments were performed on day (7 + 7).

### Preparation of neonatal rat ventricular myocytes (NRVMs)

Isolation of neonatal cardiomyocytes was performed as we previously described [19] and was approved by the Animal Experimentation Ethics Committee, the Chinese University of Hong Kong and conformed to the Guide for the Care and Use of Laboratory Animals published by the United States National Institutes of Health (NIH Publication No. 80-23, revised 2011). Neonatal male rat pups (1–2 days postnatal) were killed for NRVM isolation. The pups were rinsed quickly in 75% ethanol solution for surface sterilization. Pups were euthanized by decapitation using sterile scissors, and the chest was opened along the sternum to allow access to the chest cavity and the heart. The left ventricle was cut into 4–6 pieces and digested with 0.5 mg/mL collagenase type II (Thermo Fisher Scientific).

### Preparation of adult mouse heart

This study was approved by the Animal Experimentation Ethics Committee, the Chinese University of Hong Kong and conformed to the Guide for the Care and Use of Laboratory Animals published by the United States National Institutes of Health (NIH Publication No. 80-23, revised 2011). The mouse was euthanized. The heart was surgically removed and rinsed immediately in the ice-cold PBS. The whole mouse heart was minced into pieces and placed in the Dounce homogenizer with 1.5 mL of homogenization buffer (containing 250 mM sucrose, 10 mM Tris pH 7.4, 1 mM EDTA, and freshly supplemented with protease and phosphatase inhibitors). The heart was homogenized till the majority of the heart tissue was broken up. The homogenate was then placed in a new tube and centrifuged at 1000 g for 5 min at 4 °C to pellet the unbroken cells/debris. The supernatant was collected and stored at -80 °C for use.

### Molecular cloning

For TRPA1 knockdown, the Red-pAdTrack-U6-shTRPA1 plasmid was constructed from shTRPA1 bacterial glycerol stock (Sigma-Aldrich). shRNA targeting luciferase was used as a negative control. For TRPA1 overexpression, the Blue-pAdTrack-CMV-TRPA1 plasmid was constructed from pCI-neo-mTRPA1, which was a kind gift from Professor Yasuo Mori (Kyoto University, Japan). pAdTrack-HA-PGC-1α (Addgene) was used for PGC-1α overexpression.

### Adenovirus production and infection

The above plasmids with genes of interest were used for adenovirus packaging with the AdEasy Adenoviral Vector System Kit (Agilent Technologies, Santa Clare, CA, USA) according to the manufacturer's protocol. The concentrated adenoviruses were directly used to infect host cells. The host cells were infected for 5 h in growth media.

### Immunofluorescence staining

Primary antibodies used in the study were: anti-TRPA1 (1:200, Alomone, Jerusalem, Israel), anti-α-actinin (1:200, Abcam, Cambridge, UK). Secondary antibodies used were: Alexa Fluor 405 goat anti-mouse IgG (1:200, Alexa), Alexa Fluor 488 goat anti-mouse IgG (1:200, Thermo Fisher Scientific), Alexa Fluor 488 AffiniPure goat anti-rabbit IgG (1:200, Jackson ImmunoResearch, West Grove, PA, USA), Alexa Fluor 594 AffiniPure goat anti-mouse IgG (1:200, Jackson ImmunoResearch). Cell imaging was performed with Leica SP8 confocal microscope (Leica, Wetzlar, Germany) and analyzed by Fiji (NIH).

### Measurements of mitochondrial dynamics and biogenesis

MitoTracker Red (Thermo Fisher Scientific) was used to stain mitochondria. Images of mitochondria were segmented using the pixel classification feature of the interactive learning and segmentation toolkit software (Ilastik 1.3.0, European Molecular Biology Laboratory) and analyzed by Fiji [22]. Measures of average mitochondrial area, number, and fractional occupancy were generated using the 'analyze particles' function in Fiji. Measures of mitochondrial length, junctions (voxels with three or more neighbors), and branches (slab segments connecting endpoints to either junctions or other endpoints) were determined using the 'skeletonize' and 'analyze skeleton' plugins in Fiji.

### Mitochondrial ROS measurements

Mitochondrial ROS level was measured using CellROX Green (Thermo Fisher Scientific), which is weakly fluorescent while in a reduced state and exhibits bright green photostable fluorescence upon oxidation by ROS and subsequent binding to DNA, with absorption/emission maxima of ~ 485/520 nm. Images were acquired by Leica SP8 confocal microscope and analyzed by Fiji.

### Mitochondrial membrane potential (Ψm) measurements

Mitochondrial membrane potential (Ψm) was measured using the positively charged dye tetramethylrhodamine methyl ester (TMRE, Thermo Fisher Scientific). Images were acquired by Leica SP8 confocal microscope and analyzed by Fiji.

### Measurements of mitochondrial respiration

Mitochondrial oxidative characteristics of the mESC-CMs were tested by examining cellular oxygen consumption rates (OCR) using XF Long Chain Fatty Acid Oxidation Stress Test Kit (Agilent Technologies) with a Seahorse XF-96 Analyzer (Agilent Technologies). The data were analyzed with Agilent Seahorse Analytics (https://seahorseanalytics.agilent.com).

### Confocal Ca^2+^ imaging

Cytosol Ca^2+^ transient was measured by using Fluo-4 (Thermo Fisher Scientific). mESC-CMs were stained with 5 μM Fluo-4 at 37 °C for 15 min and observed under the Leica SP8 confocal microscope. Data were analyzed by Fiji and Excel.

### Electrophysiology

Membrane potential was measured with the ruptured whole-cell patch clamp. Microelectrodes were pulled from glass capillary (World Precision Instruments, Sarasota, FL, USA) by a pipette puller (Sutter Instrument, Novato, CA, USA) and polished with a micro forge (NARISHIGE, Japan). The microelectrodes used in experiments were typically 3–6 MΩ after they were filled with the internal solution. Both microelectrodes and solutions were prepared freshly before experiments. Axopatch 200B amplifier (Molecular Devices, Sunnyvale, CA, USA) was used to amplify the signal detected by the microelectrode. pClamp 10.4 software (Molecular Devices) was used for recording signals. Action potential (AP) data were analyzed with Cardiac Action Potential Analysis Software (CAPA) Package Distributed by Science Consulting Cardiac Cellular Electrophysiology UG (Essen, Germany) [23].

### Western blotting

Western blot was done as previously described [19–21]. Primary antibodies used in the study were as follows: anti-TRPA1 (1:1000, Alomone), anti-TRPA1 (1:1000, Novus Biologicals, CO, USA), anti-TRPA1 (1:1000, LSBio, WA, USA), MAPK Family Antibody Sampler Kit (1:1000, Cell Signaling Technology), Phospho-MAPK Family Antibody Sampler Kit (1:1000, Cell Signaling Technology), anti-MKP-1 (1:1000, Thermo Fisher Scientific), PGC-1α (1:1000, Abcam), anti-β-actin (1:1000, Abcam), anti-β-tubulin (1:1000, Cell Signaling Technology). Secondary antibodies used were: HRP-conjugated goat anti-rabbit secondary antibody (1:3000, Dako), HRP-conjugated goat anti-mouse secondary antibody (1:3000, Dako).

### Quantitative real-time PCR (qPCR) measurements

Total RNA was extracted using Trizol reagent (Thermo Fisher Scientific). Genomic DNA was removed using TURBO DNA-free DNase Treatment and Removal Reagents (Thermo Fisher Scientific). SuperScript III Reverse Transcriptase (Thermo Fisher Scientific) was used to do reverse transcription. TB Green Premix Ex Taq (Tli RNaseH Plus) (TaKaRa, Kyoto, Japan) was used for intercalator-based real-time PCR with CFX96 Real-Time PCR Detection System (Bio-Rad). Relative quantification of target gene expression was performed using the 2^−ΔΔCt^ method. Target gene expression was normalized to the housekeeping gene, and the relative gene expression of the target gene in different groups was normalized to that of the control group. Primers for qPCR were designed using Primer-Blast software by NCBI. Sequences of primers are shown in Additional file 1.

### Drugs and chemical reagents

Chembridge-5861528, TRPA1 blocker, was purchased from Alomone. Allyl isothiocyanate, TRPA1 activator, was purchased from Sigma-Aldrich. SB 203580, a specific inhibitor of p38 MAPK, was purchased from Tocris (Bristol, UK). All chemical reagents used to set up buffer solutions were purchased from Sigma-Aldrich.

### Statistical analysis

Unpaired, two-tailed Student's t-test was used for the statistical analysis using the GraphPad Prism 7 (GraphPad Software, Inc., La Jolla, CA, USA). Data were presented as mean ± SEM from at least three independent biological replicates. *P* < 0.05 was considered to indicate statistically significant differences.

## Results

### TRPA1 improved the structural maturation of mESC-CMs

The expression of TRPA1 in mESC-CMs was first examined. Western blot results showed that TRPA1 was expressed in mESC-CMs during the whole stage of differentiation and in the adult mouse heart (Additional file 2: Fig. S1A). By co-staining TRPA1 with the muscle α-actinin, TRPA1 was also shown to be present in mESC-CMs (Additional file 2: Fig. S1B). To further investigate the functional role of TRPA1 on mESC-CMs, adenoviruses with shTRPA1 or TRPA1 cDNA were used for knockdown or overexpression of TRPA1, respectively; qPCR and western blot were performed to confirm the genetic manipulation efficiency in CMs with TRPA1 knockdown (TRPA1 KD-CMs) and TRPA1 overexpression (TRPA1 OE-CMs) (Additional file 2: Fig. S1C–F).

**Fig. 1:**
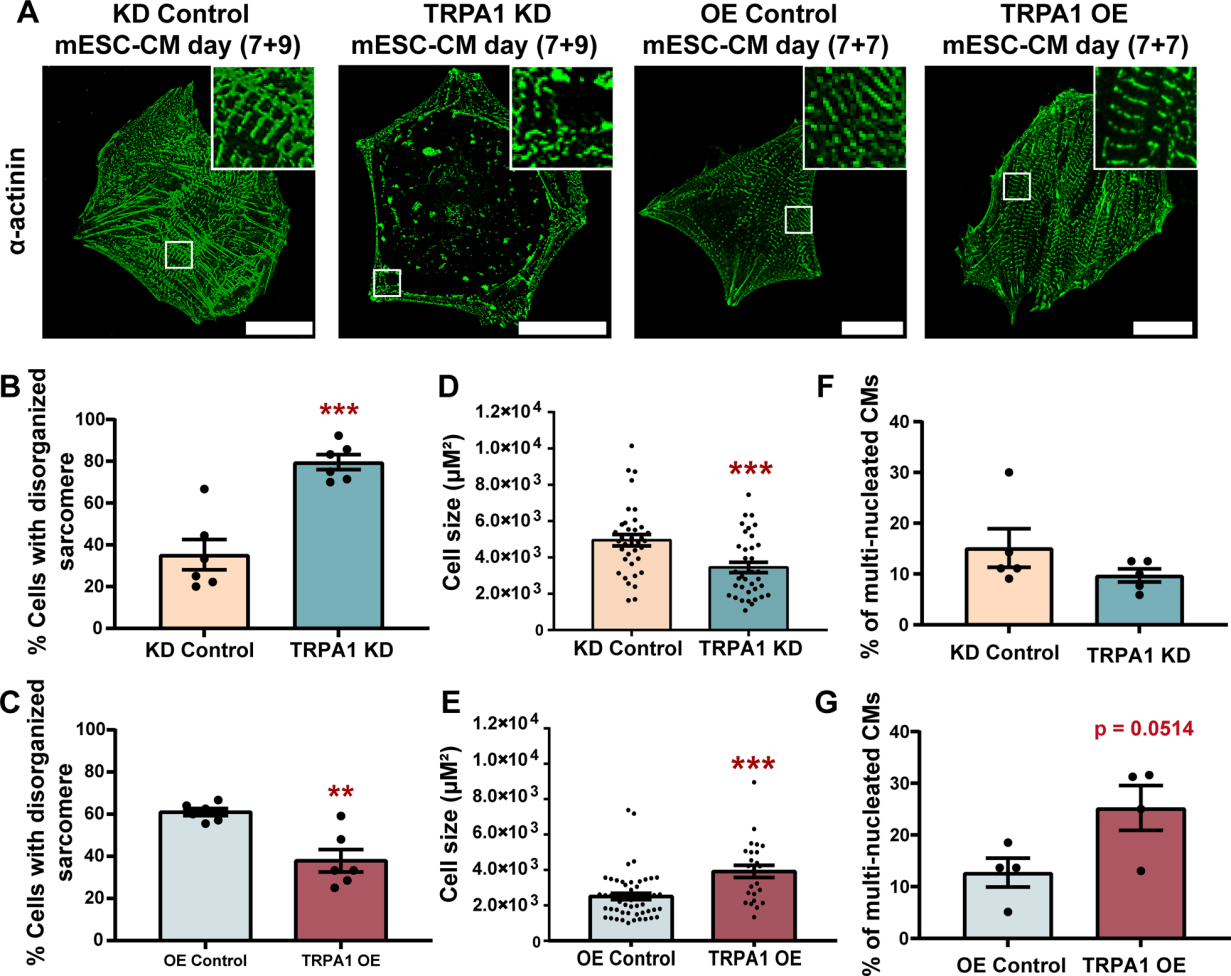
TRPA1 improved the structural maturation of mESC-CMs. **A** Representative confocal fluorescence images showing the staining of α-actinin. Scale bar = 25 μm. **B**, **C** Percentage of cells with disorganized sarcomeres for KD control, TRPA1 KD, OE control, and TRPA1 OE mESC-CMs. Data were from 6 independent batches of differentiation. **D**, **E** Cell size for KD control, TRPA1 KD, OE control, and TRPA1 OE mESC-CMs. *n* = 25–52 cells from 3 independent batches of differentiation. **F**–**G** Percentage of multi-nucleated cells in KD control, TRPA1 KD, OE control, and TRPA1 OE mESC-CMs. Data were from 4 to 5 independent batches of differentiation. Data were presented as mean ± SEM. **P < 0.01; ***P < 0.001

Knockdown of TRPA1 increased the ratio of CMs with disorganized sarcomeric organization compared with the control condition (Fig. 1A, B). On the contrary, overexpression of TRPA1 decreased the percentage of CMs with disorganized sarcomere organization compared with the control condition (Fig. 1A, C). Cell size is an important parameter for CMs because it influences impulse propagation, maximal rate of AP depolarization, as well as total contractile force [24]. Compared to control CMs, TRPA1 knockdown significantly decreased, while TRPA1 overexpression significantly increased the size of CMs (Fig. 1D, E). The percentage of multi-nucleated cells tended to decrease by TRPA1 knockdown and increase by TRPA1 overexpression (Fig. 1F, G). All these results suggested that TRPA1 improved the structural maturation of mESC-CMs.

### TRPA1 improved Ca^2+^ handling and electrophysiology properties of mESC-CMs

**Fig. 2:**
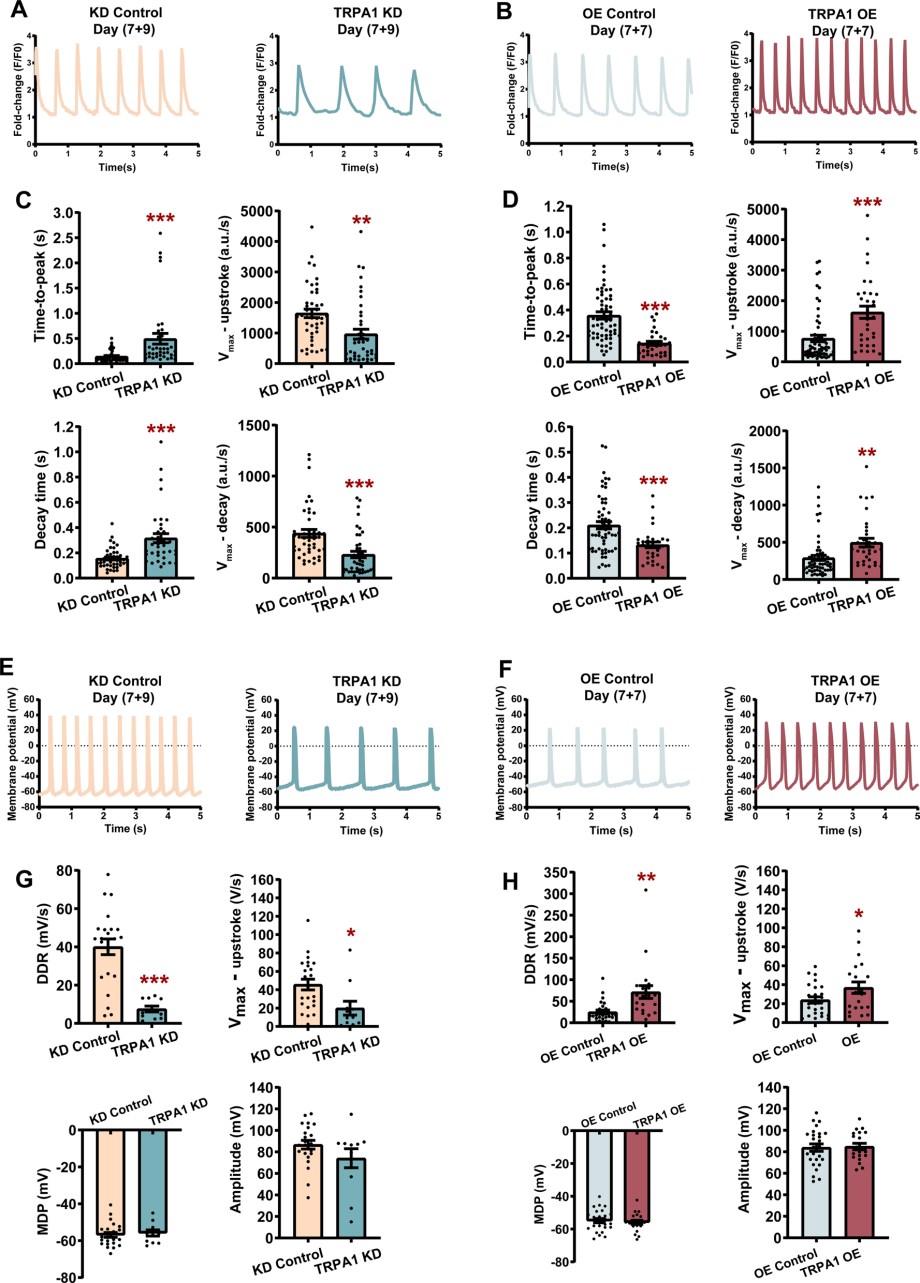
TRPA1 improved the Ca^2+^ handling and electrophysiological properties of mESC-CMs. **A**, **B** Representative calcium transients of KD control, TRPA1 KD, OE control, and TRPA1 OE mESC-CMs. **C**, **D** Bar graphs showing the time-to-peak, *V*_max_-upstroke, decay time, and *V*_max_-decay of calcium transients. *n* = 32–60 cells from at least 3 independent batches of differentiation. **E**, **F** Representative spontaneous AP tracings of KD control, TRPA1 KD, OE control, and TRPA1 OE mESC-CMs. **G**, **H** Bar graphs showing the DDR, *V*_max_-upstroke, MDP, and amplitude of APs. *n* = 11–26 cells from at least 3 independent batches of differentiation. Data were presented as mean ± SEM. *P < 0.05; **P < 0.01; ***P < 0.001

Ca^2+^ is a critical second messenger that participates in the ECC and relaxation of the heart, and involves in key signal transduction pathways in cardiac function. Regulation of contractility of CMs is achieved by a spatially defined program of ion channels, pumps, and exchangers that accurately control Ca^2+^ entry into and out of the cell and the sarcoplasmic reticulum (SR) [25]. To test whether TRPA1 is involved in Ca^2+^ handling in mESC-CMs, Ca^2+^ imaging was performed to record the cytosolic Ca^2+^ in mESC-CMs. Inhibition of TRPA1 by TRPA1 inhibitor Chembridge-5861528 (CHEM) increased the time-to-peak while the activation of TRPA1 by TRPA1 activator allyl isothiocyanate (AITC) decreased the time-to-peak and decay time of calcium transients (Additional file 2: Fig. S2A–E). The role of TRPA1 on Ca^2+^ handling was further confirmed with TRPA1 knockdown and overexpression. When compared with control CMs, TRPA1 KD-CMs exhibited increased time-to-peak and decay time. Consistently, the *V*_max_-upstroke and *V*_max_-decay were significantly decreased by TRPA1 knockdown (Fig. 2A, C). In contrast, TRPA1 OE-CMs showed decreased time-to-peak and decay time, and increased *V*_max_-upstroke and *V*_max_-decay (Fig. 2B, D).

In addition to Ca^2+^ handling, whether TRPA1 would modulate the maturity of the electrophysiological properties of mESC-CMs was investigated. Whole-cell patch clamp was conducted; the results showed that TRPA1 blocker significantly reduced while TRPA1 activator significantly increased the diastolic depolarization rate (DDR) of APs compared with control CMs (Additional file 2: Fig. S2F–J). The *V*_max_-upstroke of APs was decreased by TRPA1 blocker compared to control CMs, while there was no statistical difference between TRPA1 activator-treated CMs and control CMs. Importantly, TRPA1 knockdown markedly reduced the DDR and *V*_max_-upstroke of APs compared with the KD control (Fig. 2E, G). On the other hand, TRPA1 overexpression significantly increased the DDR and the *V*_max_-upstroke of APs compared with the OE control (Fig. 2F, H). There was no apparent difference in maximum diastolic potential (MDP) and amplitude between different groups. All these data indicated that TRPA1 positively regulates the cytosolic calcium transients and the electrophysiological properties of mESC-CMs.

### TRPA1 improved the metabolic maturation of mESC-CMs

**Fig. 3:**
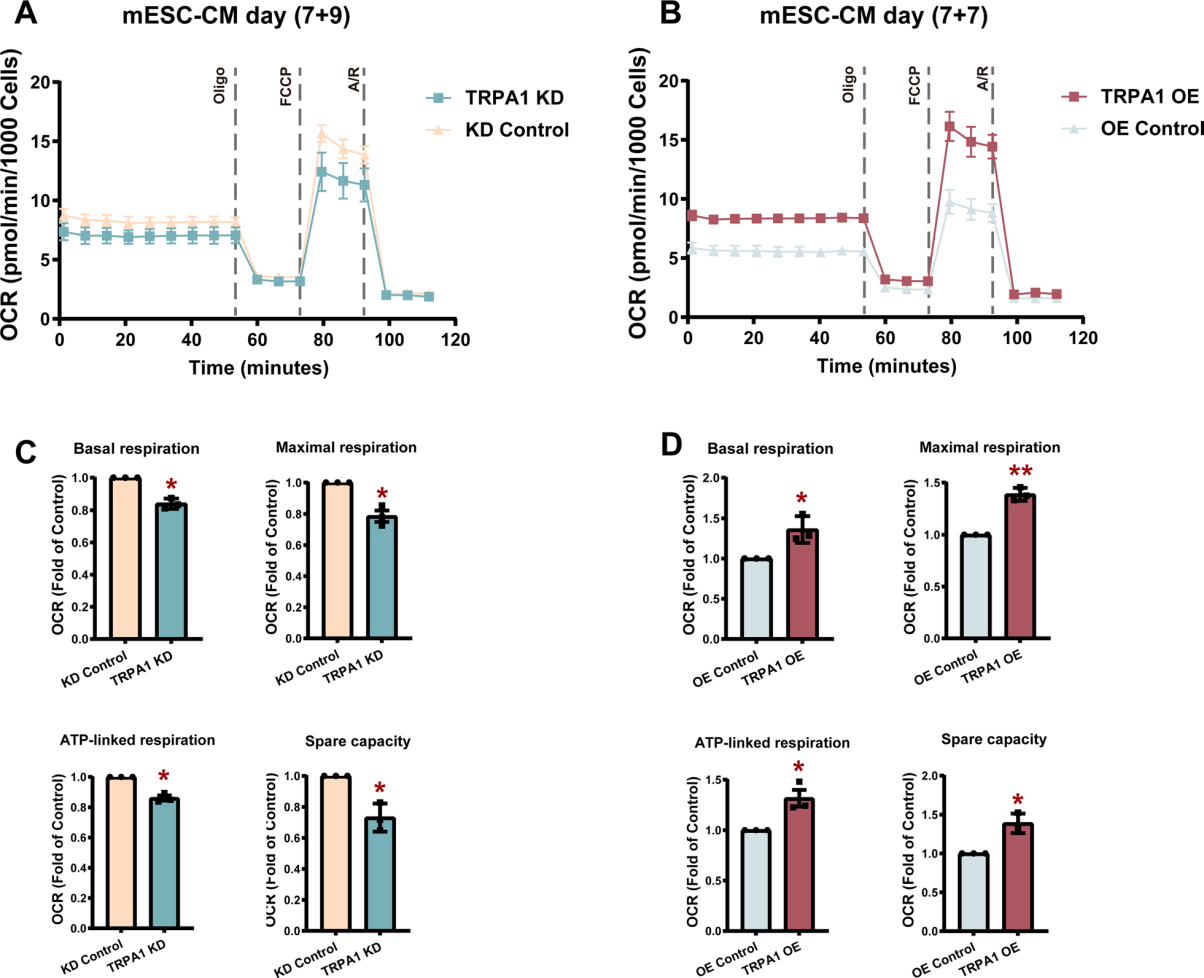
TRPA1 improved the metabolic maturation of mESC-CMs. **A**, **B** Representative real-time respiration measurements of mESC-CMs and their responses to the subsequent injection of oligomycin (oligo), carbonyl cyanide-4-(trifluoromethoxy) phenylhydrazone (FCCP), and antimycin and rotenone (A/R). **C**, **D** Bar graphs showing the basal respiration, maximal respiration, ATP-linked respiration, and spare capacity in KD control, TRPA1 KD, OE control, and TRPA1 OE mESC-CMs. Data from KD and OE groups were normalized to that of their respective control groups. Data were from three independent batches of differentiation. Data were presented as mean ± SEM. *P < 0.05; **P < 0.01

During CM maturation, the metabolism also shifts from an immature to a mature state as reflected by an increased reliance on oxidative phosphorylation for energy production. Next, we investigated whether TRPA1 affected the oxidative characteristics in mESC-CMs by examining the OCR using the Seahorse system. TRPA1 knockdown significantly reduced the basal respiration of mESC-CMs when compared with the KD control (Fig. 3A, C). Mitochondrial stress test was performed by sequentially adding oligomycin, FCCP, and antimycin/rotenone. The oligomycin injection resulted in a reduction in mitochondrial respiration linked to cellular ATP production. TRPA1 KD-CMs exhibited decreased ATP-linked respiration compared with control CMs (Fig. 3C). The FCCP-stimulated OCR was used to calculate the maximal respiration and spare respiratory capacity. TRPA1 KD-CMs demonstrated decreased maximal respiration and spare respiration compared with control CMs (Fig. 3C), indicating the repressed ability of the cells to respond to increased energy demand or under stress. On the contrary, TRPA1 OE-CMs showed elevated metabolic abilities compared with the control CMs in terms of basal respiration, ATP-linked respiration, maximal respiration, and spare capacity (Fig. 3B, D).

### TRPA1 increased mitochondrial fusion/decreased mitochondrial fission, increased mitochondrial biogenesis and function in mESC-CMs

A connected mitochondrial network is generally observed in metabolically active cells, while the mitochondria are somewhat fragmented in metabolically inactive cells [26]. To meet the increased energy demand, a robust increase in the number and size of mitochondria occurs in the heart immediately before and after birth, coinciding with the increased oxidative phosphorylation [27]. As oxidative phosphorylation occurs in mitochondria, we found that the function of TRPA1 is positively correlated with oxidative phosphorylation, we next assessed whether TRPA1 regulates the structure and biogenesis of mitochondria in mESC-CMs. MitoTracker Red staining followed by live-cell imaging was performed to examine whether mitochondrial morphology and biogenesis change upon treatment with the TRPA1 activator or blocker. Our results showed that TRPA1 blocker decreased mitochondrial size (mitochondrial area and branch length) and complexity (number of branches and junctions per mitochondrion) and increased mitochondrial number gradually, indicating an increase in mitochondrial fission/decrease in mitochondrial fusion (Additional file 2: Fig. S3A, B). On the other hand, TRPA1 activator induced the opposite effects (Additional file 2: Fig. S3A, B). Notably, the mitochondrial occupancy, which indicated mitochondrial biogenesis, did not change much with a short-term treatment with drugs. However, with the extension of treatment time, TRPA1 blocker not only resulted in more fragmented and smaller mitochondria but also significantly reduced mitochondrial occupancy, indicating changes in both mitochondrial dynamics and biogenesis (Additional file 2: Fig. S3C, D). On the other hand, TRPA1 activator induced the opposite effects (Additional file 2: Fig. S3C, D). These results suggested that short-term changes in TRPA1 activities affected mitochondrial dynamics while long-term changes in TRPA1 activities affected both mitochondrial dynamics and biogenesis.

**Fig. 4:**
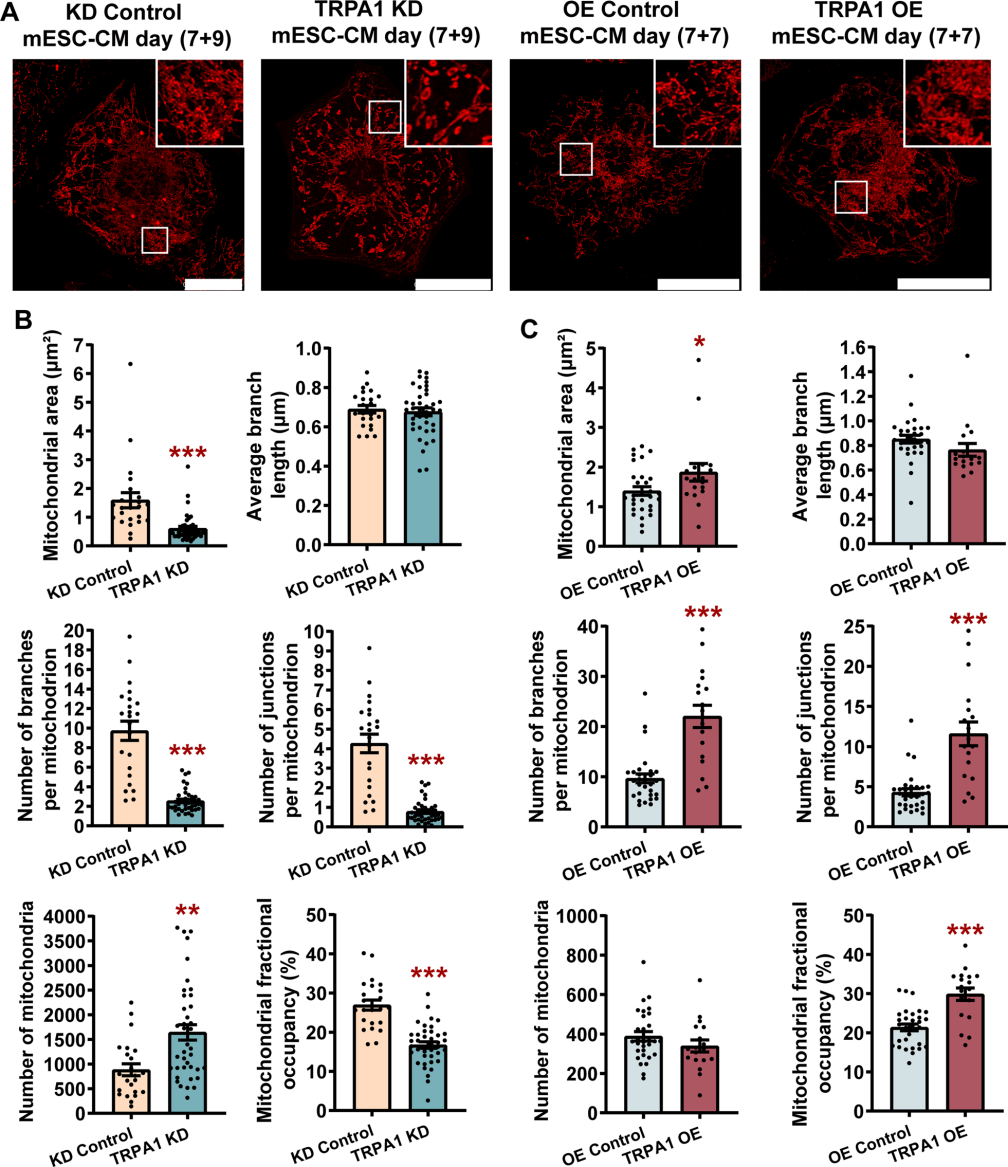
TRPA1 improved the biogenesis and fusion of mitochondria in mESC-CMs. **A** Confocal microscopy images of mitochondria in KD control, TRPA1 KD, OE control, and TRPA1 OE mESC-CMs. Scale bar = 25 μm. **B**, **C** Bar graphs showing the mitochondrial size (average mitochondrial area and branch length), mitochondrial complexity (number of branches and junctions per mitochondrion), mitochondrial number, and fractional occupancy. *n* = 15–39 cells from 3 independent batches of differentiation. Data were presented as mean ± SEM. *P < 0.05; **P < 0.01; ***P < 0.001

We further confirmed the function of TRPA1 in regulating mitochondrial dynamics and biogenesis by TRPA1 knockdown or overexpression in mESC-CMs. Our results showed that TRPA1 knockdown induced fragmented, small mitochondria while TRPA1 overexpression induced interconnected mitochondria (Fig. 4A). TRPA1 KD-CMs exhibited reduced mitochondrial area, number of branches per mitochondrion, number of junctions per mitochondrion, mitochondrial fractional occupancy, and elevated mitochondrial number compared with control CMs (Fig. 4A, B). In contrast, TRPA1 OE-CMs showed increased mitochondrial area, number of branches per mitochondrion, number of junctions per mitochondrion, and mitochondrial fractional occupancy compared with control CMs (Fig. 4A, C). There is no apparent difference in the mitochondrial number between TRPA1 OE-CMs and control CMs. Overall, our results suggested that TRPA1 negatively regulates mitochondrial fission and positively regulates mitochondrial fusion and biogenesis, which is consistent with our metabolic data.

Mitochondria are not merely the center for energy metabolism but are also the headquarters for ROS production [28]. Mitochondria are the major sites for ROS production in CMs, and the cellular ROS homeostasis is tightly regulated by mitochondria. To determine whether changes of TRPA1 activities further influenced the function of mitochondria, the level of ROS in the form of mitochondrial superoxide was measured. The results showed that mitochondrial ROS level was elevated by TRPA1 blocker and reduced by TRPA1 activator compared to the control group (Additional file 2: Fig. S4A). We next tested the mitochondrial membrane potential (Ψm), which is vital in maintaining the normal function of mitochondria. Depolarization of Ψm is commonly considered to be a prelude to irreversible apoptosis and cell death. Our result showed that the Ψm was significantly reduced (depolarized) by TRPA1 blocker and elevated (hyperpolarized) by TRPA1 activator compared with control group (Additional file 2: Fig. S4B). These data indicated that the inhibition of TRPA1 impaired the mitochondrial function while activation of TRPA1 increased the function of mitochondria.

### TRPA1 increased the expression of genes positively related to the function and maturation of mitochondria and CMs

**Fig. 5:**
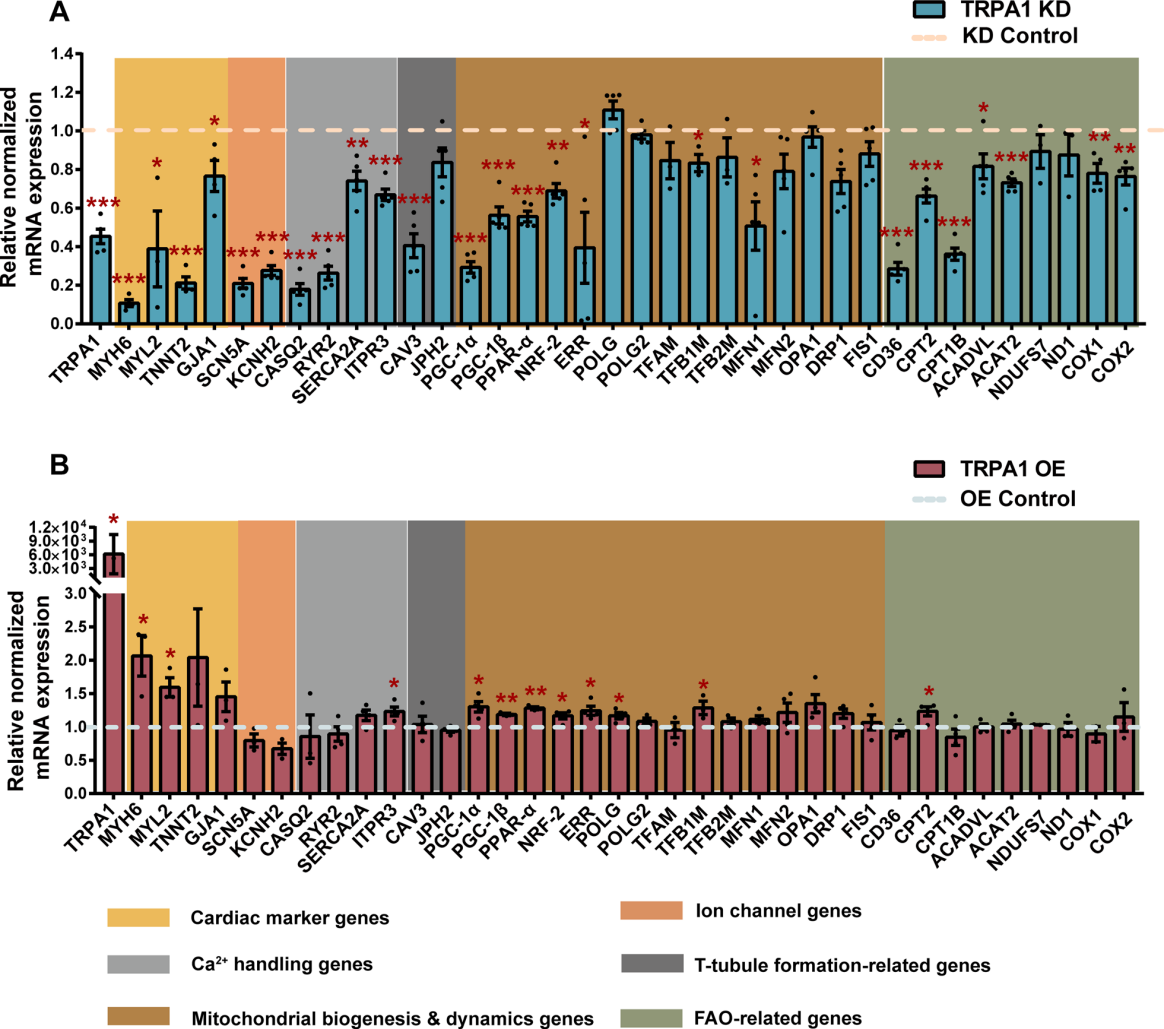
TRPA1 improved the expression of genes related to the function and maturation of mitochondria and CMs. **A**, **B** Levels of mRNA transcripts encoding TRPA1, cardiac marker genes, ion channel genes, Ca^2+^ handling genes, t-tubule formation-related genes, mitochondrial biogenesis/dynamic-related genes, and fatty acid β-oxidation (FAO)-related genes in TRPA1 KD cells (**A**) and TRPA1 OE cells (**B**). The broken lines represented the relative expression level of genes in the control groups. KD control and TRPA1 KD cells were analyzed at day (7 + 9). OE control and TRPA1 OE cells were analyzed at day (7 + 7). Data were from 4 to 5 independent batches of differentiation. Results were expressed as mean ± SEM. *P < 0.05; **P < 0.01; ***P < 0.001

The data above showed that TRPA1 could direct the structural, functional, and metabolic maturation of mESC-CMs. To further understand how TRPA1 affected CM maturation, the expressions of cardiac marker genes and key genes associated with E-C coupling, t-tubule formation, mitochondrial dynamics/biogenesis, and oxidative phosphorylation were analyzed. As shown in Fig. 5A, cardiac marker genes, *MYH6*, *MYL2*, *TNNT2*, and *GJA1* were markedly decreased by TRPA1 knockdown. The ion transporter genes *SCN5A* and *KCNH2* were significantly reduced in TRPA1 KD-CMs compared with control CMs. Calcium-handling genes, including *CASQ2*, *SERCA2a*, *RYR2*, and *ITPR3,* were also decreased in TRPA1 KD-CMs. The key players in t-tubule biogenesis, *CAV3* and *JPH2*, were suppressed by TRPA1 knockdown. In addition, TRPA1 KD-CMs exhibited reduced expression of *PGC-1α/β* as well as their downstream transcription factors (*PPAR-α*, *NRF-2*, *ERRα*). *TFB1M*, the gene essential for mitochondrial function, was also reduced by TRPA1 knockdown. Expression of the gene mediating mitochondrial fusion, *MFN1*, was downregulated in TRPA1 KD-CMs. *CD36*, a gene that regulates fatty acid uptake, was significantly reduced in TRPA1 KD-CMs. Other genes involved in fatty acid oxidation, including *CPT2*, *CPT1B*, *ACADVL*, *ACAT2*, *COX1*, and *COX2* were also repressed by TRPA1 knockdown (Fig. 5A). Conversely, overexpression of TRPA1 significantly elevated the expressions of multiple of these genes (*MYH6*, *MYL2*, *IPTR3*, *PGC1-α*, *PGC1-β*, *PPAR-α*, *NRF-2*, *ERRα, POLG*, *TFB1M*, and *CPT2*) (Fig. 5B). All these data indicated that TRPA1 participated in regulating the function and maturation of mitochondria and CMs.

### The attenuated CM maturation induced by TRPA1 knockdown could be ameliorated by PGC-1α overexpression

**Fig. 6:**
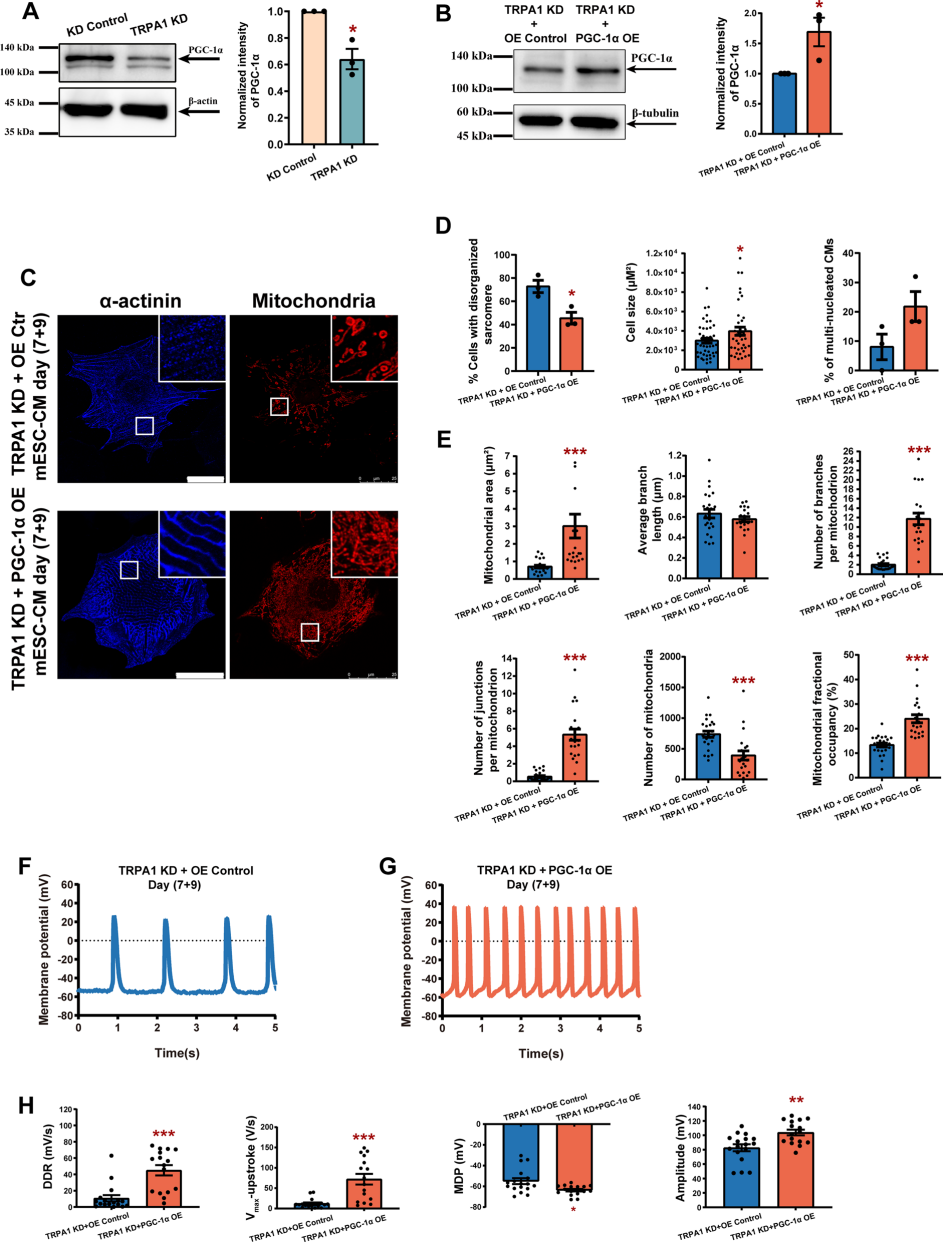
The attenuated CM maturation induced by TRPA1 knockdown could be ameliorated by PGC-1α overexpression. **A** Representative western blots and bar graph showing the expression of PGC-1α in KD control and TRPA1 KD mESC-CMs. **B** Representative western blots and bar graph showing the expression of PGC-1α in TRPA1 KD mESC-CMs with or without PGC-1α overexpression. **C** Confocal microscopy images of α-actinin and mitochondria in TRPA1 KD mESC-CMs with or without PGC-1α overexpression. Scale bar = 25 μm. **D** Bar graphs showing the percentage of cells with disorganized sarcomeres, cell size, and percentage of multi-nucleated cells in TRPA1 KD mESC-CMs with or without PGC-1α overexpression. *n* = 40–46 cells from 3 independent batches of differentiation. **E** Bar graphs showing the mitochondrial size (average mitochondrial area and branch length), mitochondrial complexity (number of branches and junctions per mitochondrion), mitochondrial number, and fractional occupancy. *n* = 21–24 cells from 3 independent batches of differentiation. **F**, **G** Representative spontaneous AP tracings of TRPA1 KD mESC-CMs with or without PGC-1α overexpression. **H** Bar graphs showing the DDR, *V*_max_-upstroke, MDP, and amplitude of APs. *n* = 16–17 cells from 3 independent batches of differentiation. Results were expressed as mean ± SEM. *P < 0.05; **P < 0.01; ***P < 0.001. Blots presented in panels (**A**) and (**B**) have been chopped. Full-length blots are presented in Additional file 3: Fig. S7

Our data have demonstrated that TRPA1 improved mitochondrial fusion and biogenesis and promoted CM maturation. Next, we investigated the mechanism underlying how TRPA1 exerts its role on CMs. As mentioned above, PGC-1α is a transcriptional coactivator that controls energy homeostasis by regulating glucose and oxidative metabolism. Consistent with data at the mRNA level (*c.f.* Figure 5A), the expression of PGC-1α proteins was downregulated in TRPA1 KD-CMs (Fig. 6A, B), suggesting that PGC-1α may be the mediator in the regulation of CM maturation by TRPA1.

We further tested whether PGC-1α overexpression could reverse the impaired maturation of CMs caused by TRPA1 knockdown. Overexpression of PGC-1α improved the maturation of CMs, showing as reduced sarcomeric disorganization, increased cell size, and enhanced electrophysiological properties compared with OE control (Additional file 2: Fig. S5A–C). Overexpression of PGC-1α in TRPA1 KD-CMs decreased the ratio of CMs with disorganized sarcomere and increased CM size compared with TRPA1 KD-CMs (Fig. 6C, D). When compared with OE control, CMs overexpressed with PGC-1α exhibited increased mitochondrial occupancy, but with similar mitochondrial area, complexity and count (Additional file 2: Fig. S5D). Overexpression of PGC-1α in TRPA1 KD-CMs significantly increased the mitochondrial area, complexity, and occupancy compared with TRPA1 KD-CMs (Fig. 6E). In addition, PGC-1α overexpression in TRPA1 KD-CMs markedly improved the electrophysiology properties hindered by TRPA1 knockdown as shown by an increase in DDR, *V*_max_-upstroke, and amplitude of AP (Fig. 6F–H). These results suggested that TRPA1 improves CM maturation via PGC-1α.

### TRPA1 regulated PGC-1α through the p38 MAPK pathway

**Fig. 7:**
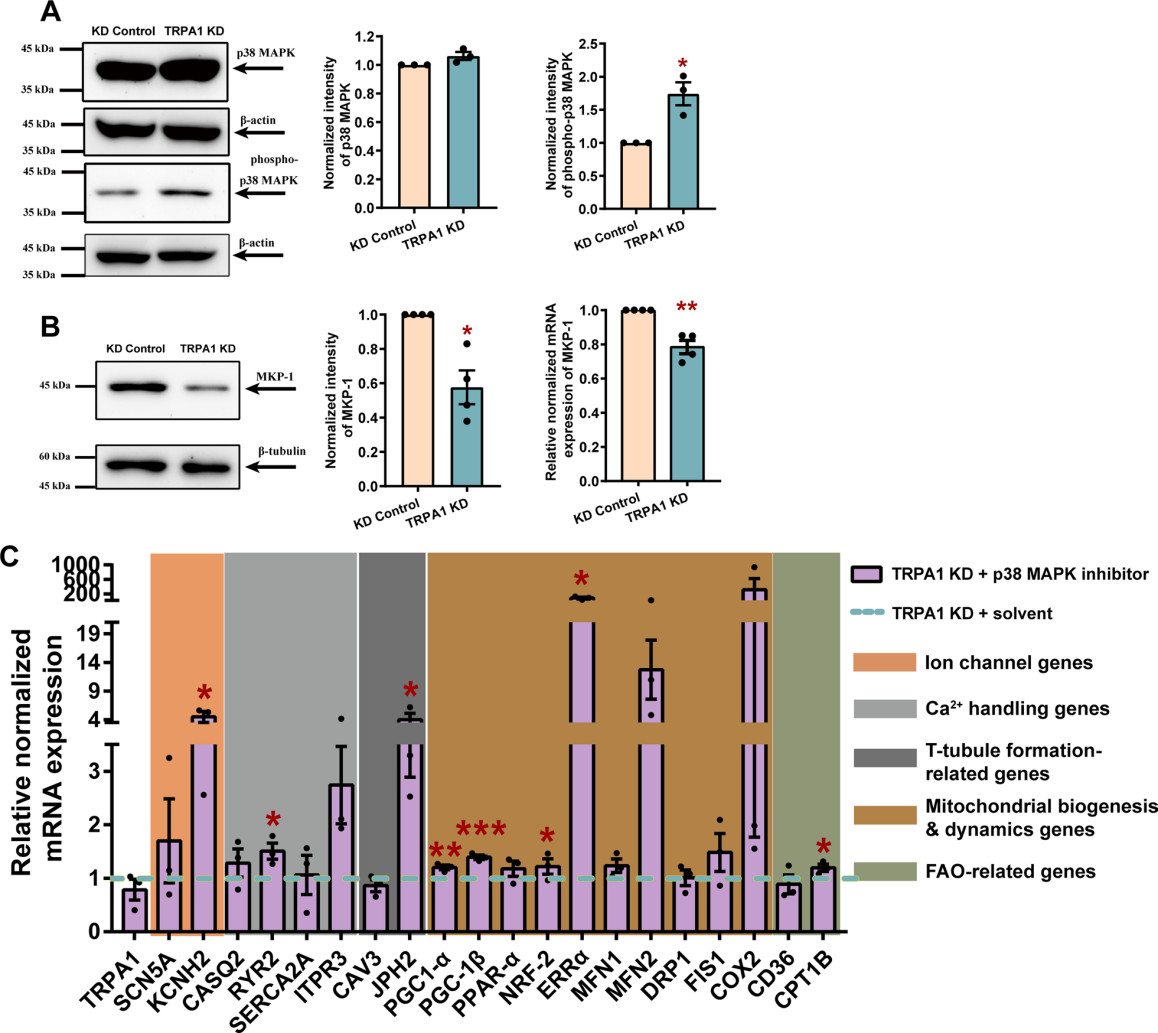
TRPA1 regulated PGC-1α through the p38 MAPK pathway. **A** Representative western blots and bar graphs showing the expression of p38 MAPK and phospho-p38 MAPK in NRVMs. **B** Representative western blots and bar graph showing the protein and mRNA expressions of MKP-1 in NRVMs. **C** Levels of mRNA transcripts encoding TRPA1, ion channels, Ca^2+^ handling genes, t-tubule formation-related genes, mitochondrial biogenesis dynamics-related genes, and fatty acid β-oxidation-related genes in TRPA1 knockdown cells with or without p38 MAPK inhibitor. Results were expressed as mean ± SEM from 3 independent isolation of NRVM or 3 batches of differentiation. *P < 0.05; **P < 0.01; ***P < 0.001. Blots presented in panels (**A**) and (**B**) have been chopped. Full-length blots are presented in Additional file 3: Fig. S7

P38 MAPK is activated by various cytokines and cellular stresses and participates in signaling cascades controlling cellular responses to cytokines and stresses. Several studies have demonstrated the relationship between activated p38 MAPK and PGC-1α, but with contradicting results. Wright et al*.* showed that activation of p38 MAPK led to an increase in PGC-1α expression and mitochondrial biogenesis in myotubes [29]. Conversely, Palomer et al*.* showed that p38 MAPK activation downregulated PGC-1α expression in CMs [30]. Our results showed that the level of phosphorylated p38 MAPK was significantly elevated by TRPA1 knockdown without apparent changes in total p38 MAPK expression (Fig. 7A), indicating increased activation of p38 MAPK. In addition, MAPK phosphatase-1 (MKP-1), which can selectively inactivate MAPKs by dephosphorylation of the regulatory threonine and tyrosine residues and has the highest degree of action against p38 MAPK [31], was markedly reduced by TRPA1 knockdown both at mRNA and protein levels (Fig. 7B). As Ca^2+^ is both necessary and sufficient for the induction of MKP-1 expression [32], our results suggested that the decreased Ca^2+^ influx from TRPA1 downregulation reduced the expression of MKP-1, further causing the unrestrained p38 MAPK activation, leading to a decrease in PGC-1α expression and mitochondrial biogenesis..

**Fig. 8:**
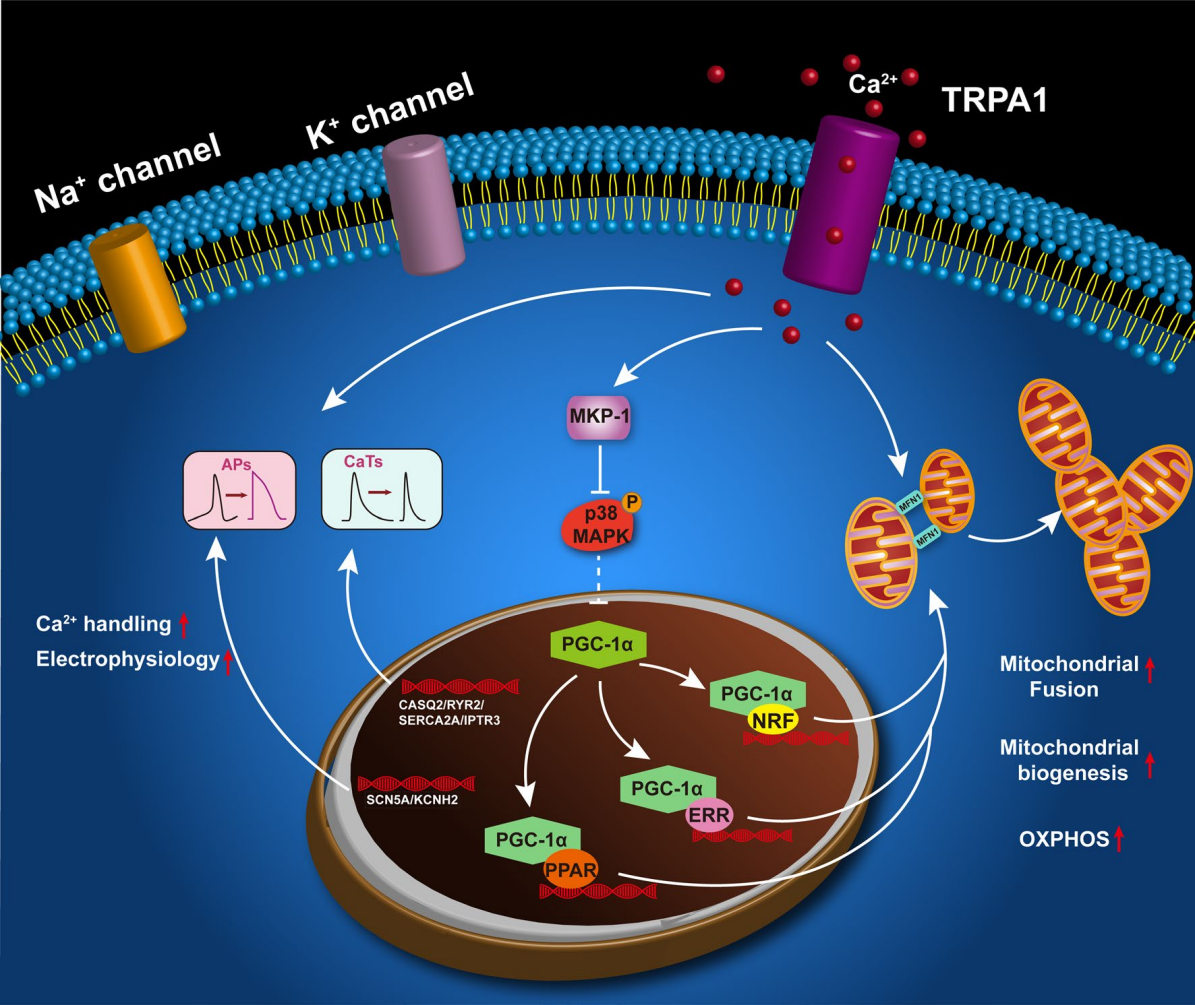
Schematic diagram showing how TRPA1 participates in the function and maturation of mESC-CMs. TPRA1 positively regulates the function and maturation of mESC-CMs via the MKP-1-p38 MAPK-PGC-1α pathway. The functional presence of TRPA1 regulates Ca^2+^ entry across the plasma membrane into the cytosol. On the one hand, the Ca^2+^ directly affects Ca^2+^ handling, electrophysiological properties, and mitochondrial dynamics. On the other hand, the increased Ca^2+^ influx through TRPA1 induces the expression of MKP-1, a Ca^2+^-promoted MAPK suppressor. MKP-1, in turn, reduces the unstrained activation of p38 MAPK, further relieving the inhibition of activated p38 MAPK on PGC-1α. The elevated expression of PGC-1α promotes mitochondrial biogenesis and CM maturation

To confirm that TRPA1 regulates the expression of PGC-1α via modulating the activity of p38 MAPK, SB 203580, a specific inhibitor of p38 MAPK, was applied to TRPA1 KD-CMs. qPCR was performed to detect the expression of genes involved in ion channels, calcium handling, t-tubule formation, mitochondrial biogenesis/ dynamics, and oxidative phosphorylation. While the knockdown of TRPA1 decreased the expression of these genes, p38 MAPK inhibitor was found to reverse the downregulation of these genes (Fig. 7C), suggesting that TRPA1 participates in CM maturation by regulating MKP-1-p38 MAPK-PGC-1α pathway (Fig. 8).

## Discussion

This study is the first study to reveal the positive role of TRPA1 channels in regulating the maturation of PSC-CMs. The generation of CMs from PSCs has provided huge promise for new therapeutic strategies to treat congenital and non-congenital heart diseases [33–35]. However, the incomplete morphological and functional maturation of PSC-CMs represents a crucial bottleneck in their utility [36]. Thus, obtaining more mature PSC-CMs is of great importance. While several previous studies have demonstrated that TRP channels participate in the development of cardiac hypertrophy and remodeling via Ca^2+^ influx [37–39], there is no study on whether TRP channels could improve cardiac maturation. Our current study revealed novel information that, functioning of TRPA1 positively regulates the maturation of ESC-CMs as assessed by the morphology, structure, calcium handling, electrophysiology, and metabolism of ESC-CMs. This study also interestingly revealed that TRPA1 improves the maturation of mESC-CMs through regulating mitochondrial biogenesis and dynamics, and the novel pathway MPK-1-MAPK- PGC-1α is involved.

Some previous studies have demonstrated that metabolic maturation promoted the functional maturation of PSC-CMs [7, 40]. Our results showed that TRPA1 positively regulates mitochondrial biogenesis and fusion; concomitantly, TRPA1 positively regulates mitochondrial oxidative phosphorylation. It is believed that both newly synthesized mitochondria and healthy mitochondria tend to fuse, because fused, interconnected mitochondria function better than fragmented mitochondria [41, 42]. We speculate that the increased mitochondrial biogenesis and fusion leads to elevated oxidative phosphorylation and metabolic maturation, which further promotes the maturation of CMs.

Expression of nuclear genome-encoded mitochondrial proteins is regulated by transcription factors and transcriptional coactivators [4]. PGC-1α and PGC-1β are the transcriptional coactivators related to mitochondrial biogenesis; in mammals, mitochondrial biogenesis is primarily regulated by the PGC-1α [43]. PGC-1α is a central regulator of nucleus-encoded mitochondrial genes through coactivation and enhancement of the expression and activity of multiple transcription factors, including NRF1, NRF2, PPARα, and ERRα [44, 45]. Interestingly, our study found that the activity of TRPA1 upregulated the expression of PGC-1α and PGC-1β and their downstream transcription factors, including PPAR-α, NRF-2, ERRα. Among them, NRF2 was known for regulating the expression of COX subunit [46] and mitochondrial transcription factors [47, 48] including TFAM and TFB isoforms (which are required for expression of genes of respiratory chain). TFB1M is known to control mitochondrial protein translation by adenine dimethylation of 12S ribosomal RNA (rRNA); loss of TFB1M induced decrease in the steady-state levels of the mitochondrially encoded respiratory chain subunits COX1 and COX2 [49]. In line with the role of TRPA1 in regulating mitochondrial biogenesis, knockdown of TRPA1 was found to downregulate TFB1M, COX1 and COX2. Importantly, while knockdown of TRPA1 negatively affected the maturation of ESC-CMs as revealed by multiple parameters at different levels, overexpression of PGC-1α reversed the effects of TRPA1 knockdown. Our results strongly suggested that TRPA1 regulated the maturation of ESC-CMs through a pathway involving PGC-1α, the master regulator of mitochondrial biogenesis and metabolism; in addition, TPRA1 regulates mitochondrial biogenesis at least at transcriptional level.

Apart from mitochondrial biogenesis, TRPA1 was also found to positively correlated to mitochondrial fusion. The study of Eisner et al*.* demonstrated that mitochondrial fusion is mediated by MFN1 and is dependent on ECC activity/RyR2-mediated [Ca^2+^]_i_ oscillations in NRVMs [50]. The study reported that the decrease of spontaneous contraction frequency decreased mitochondrial fusion, while field-stimulation of the cells increased mitochondrial fusion. It also showed that oscillatory, but not sustained calcium transients increased mitochondrial fusion, suggesting that the Ca^2+^ component of the ECC alone can regulate cardiac mitochondrial dynamics. This may help to explain why the blockage/knockdown of TRPA1 decreased mitochondrial fusion in our study. We speculate that the reduced frequency and kinetics of calcium transients caused by TRPA1 blockage/knockdown decreased mitochondrial fusion in mESC-CMs. In addition, the reduced expression of the mitochondrial fusion gene, MFN1, in TRPA1 KD-CMs also caused a decrease in mitochondrial fusion.

In line with the changes in mitochondrial biogenesis and dynamics caused by the alteration in TRPA1 expression/activity, TRPA1 was also found to regulate mitochondrial activity. Decreasing the expression/activity of TRPA1 was found to decrease the oxidative phosphorylation, increase the production of ROS and depolarize the mitochondrial membrane, while increasing the expression/ activity of TRPA1 caused the opposite effects. As the major site of ROS production, alterations in mitochondrial activity caused by disruption of mitochondrial biogenesis/dynamics are closely associated with changes in ROS levels. For example, the production of ROS increases when there is a high NADH/NAD^+^ ratio in the mitochondrial matrix. In addition, mitochondrial dehydrogenases are Ca^2+^-sensitive enzymes. Interestingly, it has been reported that cytosolic Ca^2+^ oscillations could be efficiently transduced into mitochondrial Ca^2+^ oscillations, which thereby activated these dehydrogenases [51]. A previous study showed that TRPA1 can regulate cellular and mitochondrial Ca^2+^ influx in lysophosphatidylcholine-treated human macrophages although the detailed mechanism is not elucidated [52]. Taken together the results of Ca^2+^ measurement in our current study and information from previous literature, we propose that in CMs TRPA1 regulates both cytosolic and mitochondrial Ca^2+^; the latter would in turn affect the activity of mitochondrial enzymes such as mitochondrial dehydrogenases. Subsequently, the changes in mitochondrial activity would also be reflected as changes in production of ROS. Besides, mitochondrial ROS balance and mitochondrial dynamics also interplay mutually [53]. The imbalance of ROS level may also be a result of the changes in mitochondrial dynamics caused by TRPA1.

Intriguingly, Zhu et al. demonstrated that TRPA1 protected kidneys from sepsis-related injury by promoting mitochondrial biogenesis and dynamic balance [54]. Their study revealed that mitochondrial biogenesis markers, including PGC-1α, NRF1, and TFAM, and mitochondrial fusion were decreased by TRPA1 blocker treatment in kidney tissues from either healthy or sepsis mouse. The decrease in mitochondrial biogenesis and fusion induced by TRPA1 blockage is consistent with the results observed in our study.

PGC-1α has been reported to be regulated by several pathways. The relationship between PGC-1α and p38 MAPK has long been studied, but with controversial results. Activation of the p38 MAPK pathway in skeletal muscle has been shown to promote PGC-1α expression [29]. Conversely, another study showed that TNF-α reduced PGC-1α expression through p38 MAPK activation, leading to an increased glucose oxidation and cardiac metabolism dysregulation in a human cardiac cell model [30]. In our current study, p38 MAPK was found to be activated by TRPA1 knockdown, suggesting that TRPA1 may regulate the expression of PGC-1α through negatively modulating the activity of p38 MAPK. This was verified by the experiments using p38 MAPK inhibitor; p38 MAPK inhibitor reversed the expression of genes downregulated by TRPA1 knockdown. However, how p38 MAPK regulates PGC-1α requires further studies. To investigate how TRPA1 modulates p38 MAPK, we then considered the function of MKP-1, an inhibitor of MAPKs, especially of p38 MAPK [31, 55]. A previous study showed that unrestrained p38 MAPK activity diminished cardiac contractility and Ca^2+^ handling, while MKP-1 played cardioprotective roles in the heart by dampening the activity of p38 MAPK [56]. Considering that Ca^2+^ is both necessary and sufficient for the induction of MKP-1 expression [32], we deduced that the decreased Ca^2+^ influx by TRPA1 inhibition/knockdown reduced the expression of MKP-1, further causing an unrestrained p38 MAPK activation; this would, in turn, decrease the expression of PGC-1α and mitochondrial biogenesis. Apart from its role in the pathway regulating mitochondrial biogenesis, MKP-1 was also reported to rescue diabetic nephropathy by reducing mitochondrial fission via repressing JNK-mediated mitochondrial fission factor phosphorylation [57]. However, our results revealed that TRPA1 knockdown did not affect the expression and activity of JNK and ERK (Additional file 2: Fig. S6).

As a superfamily of cation channels, different members of the TRP channel family share some structural similarities; some studies interestingly reported that the activity of one specific TRP channel can be compensated for by other members of the TRP family. For instance, TRPA1 and TRPM8 are both activated by cold temperature. TRPM8 is activated by innocuous cold (or cool, < 27 °C), icilin, menthol and a number of other odorants and cooling compounds [58], while TRPA1 is activated by noxious cold (< 18 °C), icilin (at higher concentrations than TRPM8), pungent agents such as allyl isothiocyanate (mustard oil), and other irritants [14]. A study showed that TRPM8-deficient mice have normal nociceptive-like responses to subzero centigrade temperatures, suggesting a compensatory effect by TRPA1 [59]. Similarly, inflammation-induced mechanical hyperalgesia is reduced in the TRPA1 knockdown mice, but not in the knockouts, implying the existence of a compensatory mechanism that takes over the function of the missing TRPA1 in the knockout animals and restores mechanical hyperalgesia during inflammation [60]. However, the compensatory effect of TRP channels in heart is unexplored.

In this study, we have demonstrated that TRPA1 positively regulates the maturation of ESC-CMs via regulating mitochondrial biogenesis and mitochondrial dynamics. Interestingly, from the point of view of developmental biology, the transition of mitochondria from small ones to interconnected networks is known to be vital for heart maturation and that mitophagy-mediated replacement of fetal mitochondria is reported to be essential for this transition [61]. In terms of pathophysiology, mitophagy was shown to attenuate inflammation-mediated myocardial injury [62]. In the future, it would be interesting to further explore the role of TRPA1 in cardiac physiology and pathophysiology via mitochondrial quality control (including mitophagy) [63, 64]. Taken together, our study unveiled the essential role of TRPA1 in the function and maturation of ESC-CMs. TRPA1 exerted its positive role in promoting mitochondrial maturation through mediating the Ca^2+^ influx and the downstream signaling pathway MKP-1-p38 MAPK-PGC-1α, further promotes the maturation of CMs (Fig. 8). Since multiple stimuli that can activate TRPA1 have been documented and that TRPA1-specific activators are also available, this study provides a novel and straightforward strategy in improving the maturation of PSC-CMs by activation of TRPA1.

## Supplementary Information

**Additional file 1. **Supplementary Methods, Supplementary Figure Legends, Supplementary Table 1.

**Additional file 2. **Supplementary Figures 1–6.

**Additional file 3. **Supplementary Figure 7.

## Data Availability

The datasets used and/or analyzed during the current study are available from the corresponding author on reasonable request.

## Abbreviations

AITC: Allyl isothiocyanate
AP: Action potential
CHEM: Chembridge-5861528
CMs: Cardiomyocytes
DDR: Diastolic depolarization rate
ECC: Excitation–contraction coupling
ESC-CMs: Embryonic stem cell-derived cardiomyocytes
MDP: Maximum diastolic potential
MKP-1: MAPK phosphatase-1
Ψm: Mitochondrial membrane potential
NRVM: Neonatal rat ventricular myocytes
OCR: Oxygen consumption rates
PGC-1α: Peroxisome proliferator-activated receptor gamma coactivator-1α
PSCs: Pluripotent stem cells
PSC-CMs: Pluripotent stem cell-derived cardiomyocytes
qPCR: Quantitative real-time PCR
SR: Sarcoplasmic reticulum
TMRE: Tetramethylrhodamine methyl ester
TRP: Transient receptor potential
TRPA1: Transient receptor potential ankyrin 1

## References

[1] Laflamme MA, Chen KY, Naumova AV, Muskheli V, Fugate JA, Dupras SK (2007). Cardiomyocytes derived from human embryonic stem cells in pro-survival factors enhance function of infarcted rat hearts. Nat Biotechnol.

[2] Mummery C, Ward D, van den Brink CE, Bird SD, Doevendans PA, Opthof T (2002). Cardiomyocyte differentiation of mouse and human embryonic stem cells. J Anat.

[3] Guo Y, Pu WT (2020). Cardiomyocyte maturation: new phase in development. Circ Res.

[4] Ding Q, Qi Y, Tsang SY (2021). Mitochondrial biogenesis, mitochondrial dynamics, and mitophagy in the maturation of cardiomyocytes. Cells.

[5] Yang X, Pabon L, Murry CE (2014). Engineering adolescence: maturation of human pluripotent stem cell-derived cardiomyocytes. Circ Res.

[6] Yang X, Rodriguez ML, Leonard A, Sun L, Fischer KA, Wang Y (2019). Fatty acids enhance the maturation of cardiomyocytes derived from human pluripotent stem cells. Stem Cell Rep.

[7] Feyen DAM, McKeithan WL, Bruyneel AAN, Spiering S, Hormann L, Ulmer B (2020). Metabolic maturation media improve physiological function of human iPSC-derived cardiomyocytes. Cell Rep.

[8] Yang X, Rodriguez M, Pabon L, Fischer KA, Reinecke H, Regnier M (2014). Tri-iodo-l-thyronine promotes the maturation of human cardiomyocytes-derived from induced pluripotent stem cells. J Mol Cell Cardiol.

[9] Chung S, Dzeja PP, Faustino RS, Perez-Terzic C, Behfar A, Terzic A (2007). Mitochondrial oxidative metabolism is required for the cardiac differentiation of stem cells. Nat Clin Pract Cardiovasc Med.

[10] Garbern JC, Lee RT (2021). Mitochondria and metabolic transitions in cardiomyocytes: lessons from development for stem cell-derived cardiomyocytes. Stem Cell Res Ther.

[11] Vega RB, Horton JL, Kelly DP (2015). Maintaining ancient organelles: mitochondrial biogenesis and maturation. Circ Res.

[12] Liu Y, Bai H, Guo F, Thai PN, Luo X, Zhang P (2020). PGC-1α activator ZLN005 promotes maturation of cardiomyocytes derived from human embryonic stem cells. Aging (Albany NY).

[13] Murphy SA, Miyamoto M, Kervadec A, Kannan S, Tampakakis E, Kambhampati S (2021). PGC1/PPAR drive cardiomyocyte maturation at single cell level via YAP1 and SF3B2. Nat Commun.

[14] Story GM, Peier AM, Reeve AJ, Eid SR, Mosbacher J, Hricik TR (2003). ANKTM1, a TRP-like channel expressed in nociceptive neurons, is activated by cold temperatures. Cell.

[15] Jordt SE, Bautista DM, Chuang HH, McKemy DD, Zygmunt PM, Högestätt ED (2004). Mustard oils and cannabinoids excite sensory nerve fibres through the TRP channel ANKTM1. Nature.

[16] Bautista DM, Jordt SE, Nikai T, Tsuruda PR, Read AJ, Poblete J (2006). TRPA1 mediates the inflammatory actions of environmental irritants and proalgesic agents. Cell.

[17] Materazzi S, Nassini R, Andrè E, Campi B, Amadesi S, Trevisani M (2008). Cox-dependent fatty acid metabolites cause pain through activation of the irritant receptor TRPA1. Proc Natl Acad Sci U S A.

[18] Wang Z, Ye D, Ye J, Wang M, Liu J, Jiang H (2019). The TRPA1 channel in the cardiovascular system: promising features and challenges. Front Pharmacol.

[19] Liu X, Zhao R, Ding Q, Yao X, Tsang SY (2021). TRPC7 regulates the electrophysiological functions of embryonic stem cell-derived cardiomyocytes. Stem Cell Res Ther.

[20] Zhao R, Liu X, Qi Z, Yao X, Tsang SY (2021). TRPV1 channels regulate the automaticity of embryonic stem cell-derived cardiomyocytes through stimulating the Na(+)/Ca(2+) exchanger current. J Cell Physiol.

[21] Qi Z, Wang T, Chen X, Wong CK, Ding Q, Sauer H (2021). Extracellular and intracellular angiotensin II regulate the automaticity of developing cardiomyocytes via different signaling pathways. Front Mol Biosci.

[22] Moore AS, Wong YC, Simpson CL, Holzbaur EL (2016). Dynamic actin cycling through mitochondrial subpopulations locally regulates the fission–fusion balance within mitochondrial networks. Nat Commun.

[23] Thieleczek R, Chang-Liao M-L, Zimmernann H-W, Wettwer E (2016). Automated cardiac action potential analysis (CAPA). Acta Physiol.

[24] Spach MS, Heidlage JF, Barr RC, Dolber PC (2004). Cell size and communication: role in structural and electrical development and remodeling of the heart. Heart Rhythm.

[25] Eisner DA, Caldwell JL, Kistamas K, Trafford AW (2017). Calcium and excitation-contraction coupling in the heart. Circ Res.

[26] Collins TJ, Berridge MJ, Lipp P, Bootman MD (2002). Mitochondria are morphologically and functionally heterogeneous within cells. Embo J.

[27] Marin-Garcia J, Ananthakrishnan R, Goldenthal MJ (2000). Heart mitochondrial DNA and enzyme changes during early human development. Mol Cell Biochem.

[28] van der Giezen M, Tovar J (2005). Degenerate mitochondria. EMBO Rep.

[29] Wright DC, Geiger PC, Han DH, Jones TE, Holloszy JO (2007). Calcium induces increases in peroxisome proliferator-activated receptor gamma coactivator-1alpha and mitochondrial biogenesis by a pathway leading to p38 mitogen-activated protein kinase activation. J Biol Chem.

[30] Palomer X, Alvarez-Guardia D, Rodríguez-Calvo R, Coll T, Laguna JC, Davidson MM (2009). TNF-alpha reduces PGC-1alpha expression through NF-kappaB and p38 MAPK leading to increased glucose oxidation in a human cardiac cell model. Cardiovasc Res.

[31] Keyse SM (1995). An emerging family of dual specificity MAP kinase phosphatases. Biochim Biophys Acta.

[32] Scimeca JC, Servant MJ, Dyer JO, Meloche S (1997). Essential role of calcium in the regulation of MAP kinase phosphatase-1 expression. Oncogene.

[33] Neef K, Drey F, Lepperhof V, Wahlers T, Hescheler J, Choi YH (2021). Co-transplantation of mesenchymal stromal cells and induced pluripotent stem cell-derived cardiomyocytes improves cardiac function after myocardial damage. Front Cardiovasc Med.

[34] Silver SE, Barrs RW, Mei Y (2021). Transplantation of human pluripotent stem cell-derived cardiomyocytes for cardiac regenerative therapy. Front Cardiovasc Med.

[35] Kobayashi H, Ichimura H, Ohashi N, Shiba Y (2021). Transplantation of pluripotent stem cell-derived cardiomyocytes into a myocardial infarction model of cynomolgus monkey. Methods Mol Biol.

[36] Liew LC, Ho BX, Soh BS (2020). Mending a broken heart: current strategies and limitations of cell-based therapy. Stem Cell Res Ther.

[37] Freichel M, Berlin M, Schürger A, Mathar I, Bacmeister L, Medert R, Emir TLR (2017). TRP channels in the heart. Neurobiology of TRP channels.

[38] Numaga-Tomita T, Nishida M (2020). TRPC channels in cardiac plasticity. Cells.

[39] Eder P, Molkentin JD (2011). TRPC channels as effectors of cardiac hypertrophy. Circ Res.

[40] Hu D, Linders A, Yamak A, Correia C, Kijlstra JD, Garakani A (2018). Metabolic maturation of human pluripotent stem cell-derived cardiomyocytes by inhibition of HIF1alpha and LDHA. Circ Res.

[41] Bach D, Pich S, Soriano FX, Vega N, Baumgartner B, Oriola J (2003). Mitofusin-2 determines mitochondrial network architecture and mitochondrial metabolism. A novel regulatory mechanism altered in obesity. J Biol Chem.

[42] Pich S, Bach D, Briones P, Liesa M, Camps M, Testar X (2005). The Charcot-Marie-Tooth type 2A gene product, Mfn2, up-regulates fuel oxidation through expression of OXPHOS system. Hum Mol Genet.

[43] Friedman JR, Nunnari J (2014). Mitochondrial form and function. Nature.

[44] Wu Z, Puigserver P, Andersson U, Zhang C, Adelmant G, Mootha V (1999). Mechanisms controlling mitochondrial biogenesis and respiration through the thermogenic coactivator PGC-1. Cell.

[45] Ventura-Clapier R, Garnier A, Veksler V (2008). Transcriptional control of mitochondrial biogenesis: the central role of PGC-1alpha. Cardiovasc Res.

[46] Virbasius JV, Virbasius CA, Scarpulla RC (1993). Identity of GABP with NRF-2, a multisubunit activator of cytochrome oxidase expression, reveals a cellular role for an ETS domain activator of viral promoters. Genes Dev.

[47] Virbasius JV, Scarpulla RC (1994). Activation of the human mitochondrial transcription factor A gene by nuclear respiratory factors: a potential regulatory link between nuclear and mitochondrial gene expression in organelle biogenesis. Proc Natl Acad Sci U S A.

[48] Gleyzer N, Vercauteren K, Scarpulla RC (2005). Control of mitochondrial transcription specificity factors (TFB1M and TFB2M) by nuclear respiratory factors (NRF-1 and NRF-2) and PGC-1 family coactivators. Mol Cell Biol.

[49] Metodiev MD, Lesko N, Park CB, Cámara Y, Shi Y, Wibom R (2009). Methylation of 12S rRNA is necessary for in vivo stability of the small subunit of the mammalian mitochondrial ribosome. Cell Metab.

[50] Eisner V, Cupo RR, Gao E, Csordas G, Slovinsky WS, Paillard M (2017). Mitochondrial fusion dynamics is robust in the heart and depends on calcium oscillations and contractile activity. Proc Natl Acad Sci U S A.

[51] Hajnóczky G, Robb-Gaspers LD, Seitz MB, Thomas AP (1995). Decoding of cytosolic calcium oscillations in the mitochondria. Cell.

[52] Tian C, Huang R, Tang F, Lin Z, Cheng N, Han X (2020). Transient receptor potential ankyrin 1 contributes to lysophosphatidylcholine-induced intracellular calcium regulation and THP-1-derived macrophage activation. J Membr Biol.

[53] Ježek J, Cooper KF, Strich R (2018). Reactive oxygen species and mitochondrial dynamics: the yin and yang of mitochondrial dysfunction and cancer progression. Antioxidants (Basel).

[54] Zhu J, Zhang S, Geng Y, Song Y (2018). Transient receptor potential ankyrin 1 protects against sepsis-induced kidney injury by modulating mitochondrial biogenesis and mitophagy. Am J Transl Res.

[55] Caunt CJ, Keyse SM (2013). Dual-specificity MAP kinase phosphatases (MKPs): shaping the outcome of MAP kinase signalling. FEBS J.

[56] Auger-Messier M, Accornero F, Goonasekera SA, Bueno OF, Lorenz JN, van Berlo JH (2013). Unrestrained p38 MAPK activation in Dusp1/4 double-null mice induces cardiomyopathy. Circ Res.

[57] Sheng J, Li H, Dai Q, Lu C, Xu M, Zhang J (2019). DUSP1 recuses diabetic nephropathy via repressing JNK-Mff-mitochondrial fission pathways. J Cell Physiol.

[58] McKemy DD, Neuhausser WM, Julius D (2002). Identification of a cold receptor reveals a general role for TRP channels in thermosensation. Nature.

[59] Dhaka A, Murray AN, Mathur J, Earley TJ, Petrus MJ, Patapoutian A (2007). TRPM8 is required for cold sensation in mice. Neuron.

[60] Kaneko Y, Szallasi A (2014). Transient receptor potential (TRP) channels: a clinical perspective. Br J Pharmacol.

[61] Gong G, Song M, Csordas G, Kelly DP, Matkovich SJ, Dorn GW (2015). Parkin-mediated mitophagy directs perinatal cardiac metabolic maturation in mice. Science.

[62] Wang Y, Jasper H, Toan S, Muid D, Chang X, Zhou H (2021). Mitophagy coordinates the mitochondrial unfolded protein response to attenuate inflammation-mediated myocardial injury. Redox Biol.

[63] Zhou H, Ren J, Toan S, Mui D (2021). Role of mitochondrial quality surveillance in myocardial infarction: from bench to bedside. Ageing Res Rev.

[64] Wang J, Zhou H (2020). Mitochondrial quality control mechanisms as molecular targets in cardiac ischemia-reperfusion injury. Acta Pharm Sin B.

