# Supplementary material for: TRPA1 promotes the maturation of embryonic stem cell-derived cardiomyocytes by regulating mitochondrial biogenesis and dynamics
Source: Stem Cell Res Ther. 2023 Jun 7;14:158. doi: 10.1186/s13287-023-03388-3 (PMC10249273; doi:10.1186/s13287-023-03388-3)
Supplement: Supplementary file 1 — Additional file 1. Supplementary Methods, Supplementary Figure Legends, Supplementary Table 1. [file 13287_2023_3388_MOESM1_ESM.docx]

**SUPPLEMENTARY METHODS**

**mESC culture and cardiac differentiation**

mESC line D3 (ATCC, Manassas, VA, USA) was maintained in an undifferentiated state on irradiated mouse embryonic fibroblasts (MEF) as previously described [1-3] where isolation of MEFs was approved by the Animal Experimentation Ethics Committee, the Chinese University of Hong Kong and conformed to Guide for the Care and Use of Laboratory Animals published by the United States National Institutes of Health (NIH Publication No. 80-23, revised 2011). The mESCs were cultured in an undifferentiation medium which contained DMEM supplemented with 15% heat-inactivated FBS (Hyclone, GE Healthcare, South Logan, UT, USA), 0.1 mM β-mercaptoethanol (Sigma-Aldrich, Darmstadt, Germany), 2 mM L-glutamine, 0.1 mM non-essential amino acids, 1% v/v penicillin-streptomycin and 1000 U/mL leukemia inhibitory factor (Chemicon, Millipore, Billerica, MA, USA). The medium was changed daily and cells were passaged every two days using 0.05% trypsin. Differentiation of mESCs into CMs was performed by the hanging drop method as previously described [1-3]. Briefly, on the day of differentiation (day 0), mESCs were detached with 0.05% trypsin and resuspended in the differentiation medium, which had the same components as the undifferentiation medium except for LIF. Embryoid body (EB) was formed in hanging drop by making each drop with 800 mESCs in 20 μL media. mESCs were differentiated from this day. On day 2, EBs were washed into a 90 mm petri dish (Thermo Fisher Scientific) with 10 mL differentiation medium. EBs were in suspension state until day 7. Then the EBs were seeded on a culture dish (Greiner bio-one, Frickenhausen, Germany) pre-coated with 0.1% gelatin (Sigma-Aldrich). Usually, spontaneously beating EBs, representing the appearance of CMs, appear one day after the attachment.

**Isolation of mESC-CMs**

On day (7 + 4), mESC-CMs were isolated as previously described [1-3]. Beating regions of EBs were isolated using a 1 mL syringe with a 27-G needle under a dissection microscope. Dissected regions were collected by centrifugation at 2800 rpm, 4°C for 5 minutes. Cell pellets were washed with PBS and digested in 500 μL NB solution which contained 0.1mg/mL collagenase B (Sigma-Aldrich), 120.0 mM NaCl, 5.4 mM KCl, 5.0 mM MgSO_4_, 5.0 mM Na Pyruvate, 20.0 mM glucose, 20.0 mM taurine, 10.0 mM HEPES and 30.0 μM CaCl_2_. The whole mixture was incubated at 37 °C, shaking at 150 rpm for 30 minutes. After that, the cells were centrifuged at 2800 rpm, 4 °C for 5 minutes. Then the cell pellets were resuspended in KB solution containing 85.0 mM KCl, 30.0 mM K_2_HPO_4_, 5.0 mM MgSO_4_, 1.0 mM EGTA, 5.0 mM pyruvic acid, 5.0 mM creatine, 20.0 mM taurine, 20.0 mM glucose, and 87 μM Na-ATP, followed by gently rotating at room temperature for 30 minutes for recovery of cells. After recovery, the cells were plated on 12 mm cover glass (Thermo Fisher Scientific) in a 24-well plate or confocal dishes (MatTek, Ashland, MA, USA) that have been pre-coated with 20 μg/mL laminin (Thermo Fisher Scientific) in 0.1% gelatin in 37 °C, 5% CO_2_ incubator. 40 minutes later, the normal differentiation medium was added to the cells for subsequent culture.

TRPA1 activator or blocker treatment experiments were performed on day (7 + 5). For experiments involving TRPA1 knockdown or TRPA1 knockdown concomitant with PGC-1α overexpression, mESC-CMs were infected with adenoviruses on day (7 + 5) and the experiments were performed on day (7 + 9). For TRPA1 or PGC-1α overexpression experiments, mESC-CMs were infected with adenoviruses on day (7 + 5) and the experiments were performed on day (7 + 7).

**Preparation of neonatal rat ventricular myocytes (NRVMs)**

Isolation of neonatal cardiomyocytes were performed as we previously described [1] and was approved by the Animal Experimentation Ethics Committee, the Chinese University of Hong Kong and conformed to Guide for the Care and Use of Laboratory Animals published by the United States National Institutes of Health (NIH Publication No. 80-23, revised 2011). Neonatal male rat pups (1-2 days postnatal) were sacrificed for NRVM isolation. The pups were rinsed quickly in 75% ethanol solution for surface sterilization. Pups were euthanized by decapitation using sterile scissors, and the chest was opened along the sternum to allow access to the chest cavity and the heart. Then the heart was taken out and washed with cold PBS. The left ventricle was cut into 4-6 pieces and digested with 0.5mg/mL collagenase type II (Thermo Fisher Scientific) with gentle agitation for 10 minutes. The digestion reaction was stopped by DF20 containing DMEM/F12 medium (Thermo Fisher Scientific) and 20% FBS (Thermo Fisher Scientific). The digestion step was repeated for three times. The dissociated cells were centrifuged at 1500 rpm at 4 ℃ for 10 minutes. Cell pellets were resuspended with the plating medium containing DMEM/F12 medium, 5% FBS, 10% horse serum (Thermo Fisher Scientific), and 1% v/v penicillin-streptomycin and plated into 100 mm culture dishes pre-coated with 0.1% gelatin for 30 minutes to remove fibroblasts. The suspended CMs were collected and plated into culture dishes for further use.

**Molecular cloning**

For TRPA1 knockdown, Red-pAdTrack-U6-shTRPA1 plasmid was constructed from shTRPA1 bacterial glycerol stock (Sigma-Aldrich). shRNA targeting luciferase was used as a negative control. For TRPA1 overexpression, the Blue-pAdTrack-CMV-TRPA1 plasmid was constructed from pCI-neo-mTRPA1, which was a kind gift from Professor Yasuo Mori (Kyoto University, Japan). pAdTrack-HA-PGC-1α (Addgene) was used for PGC-1α overexpression.

**Adenovirus production and infection**

The above plasmids with genes of interest were used for adenovirus packaging with the AdEasy Adenoviral Vector System Kit (Agilent Technologies, Santa Clare, CA, USA) according to the manufacturer's protocol. Briefly, the plasmid DNA was linearized using PmeI (NEB) and transformed into BJ5138 electroporation competent cells (Agilent Technologies, Santa Clara, CA, USA) which contained pAdEasy-1 vector to produce recombinant adenovirus plasmid by electroporation method. After electroporation, the bacteria were plated onto LB-kanamycin plates and incubated at 37 °C for 24 hours. The next day, several smallest, well-isolated colonies were picked from the plate and shaked in 7 mL LB-kanamycin broth at 37 °C overnight. Then, the plasmid DNA was isolated and confirmed by restriction digestion with PacI (NEB).

Positive recombinant adenovirus plasmids were used to transform *E. coli* DH5α for further amplification. AD-293 cell line was used for adenovirus packaging. AD-293 cells were plated at 50% confluent per T75 flask in the growth medium (with compositions same as MEF medium) 24 hours before the transfection. The cells should be approximately 70% confluent when transfection is conducted. For each transfection, 24 μg of recombinant adenovirus plasmid DNA was digested with PacI and transfected into AD-293 cells with Lipofectamine 2000 (Thermo Fisher Scientific). Then the cells were incubated for 7-10 days. The progress of the transfection was monitored by fluorescence microscopy. After 7-10 days of infection, the cells were collected to extract adenoviruses by repeated freeze-and-thaw. The primary adenoviruses were further amplified in AD-293 cells two more times to gain a higher titration.

The concentrated adenoviruses were directly used to infect host cells. The host cells were infected for 5 hours in growth media.

**Immunofluorescence staining**

mESC-CMs on cover glass or confocal dish were fixed with 4% paraformaldehyde (Sigma-Aldrich) (dissolved in PBS) for 10 minutes and subsequently permeabilized with 0.1% Triton X-100 (Sigma-Aldrich) for 10 minutes. Then the cells were incubated in the blocking solution containing 0.5% non-fat dry milk (Bio-Rad, Hercules, CA, USA) and 5% normal goat serum (Thermo Fisher Scientific) in PBS for 1 hour. After that, the cells were incubated with primary antibodies at 4 °C overnight. The next day, the cells were washed four times with 0.1% tween-20 (Sigma-Aldrich) (PBST) and incubated with fluorophore-conjugated secondary antibodies for 1 hour at room temperature. After incubating secondary antibodies, the cells were washed with PBST four times with gentle shaking. mESC-CMs on cover glass were mounted with fluorescence mounting medium (S302380, Dako, Copenhagen, Denmark). After placing at room temperature for at least 1 hour, the edge of the cover glass was sealed with transparent nail polish.

Primary antibodies used in the study were: anti-TRPA1 (1:200, Alomone, Jerusalem, Israel), anti-α-actinin (1:200, Abcam, Cambridge, UK). Secondary antibodies used were: Alexa Fluor 405 goat anti-mouse IgG (1:200, Alexa), Alexa Fluor 488 goat anti-mouse IgG (1:200, Thermo Fisher Scientific), Alexa Fluor 488 AffiniPure goat anti-rabbit IgG (1:200, Jackson ImmunoResearch, West Grove, PA, USA), Alexa Fluor 594 AffiniPure goat anti-mouse IgG (1:200, Jackson ImmunoResearch). Cell imaging was performed with Leica SP8 confocal microscope (Leica, Wetzlar, Germany). Cells were imaged with oil-immersion 63× objective using the 405 nm laser and 450/50 filter for DAPI or BFP, 488 nm and 525/20 for GFP, 572 nm and 593/40 for RFP.

**Measurements of mitochondrial dynamics and biogenesis**

For live-cell imaging, mESC-CMs were stained with 20 nM MitoTracker Red (Thermo Fisher Scientific) in normal differentiation media for 30 minutes at 37 °C in a 5% CO_2_ incubator. After staining, the cells were washed with and incubated in Tyrode’s solution [140 mM NaCl, 5.4 mM KCl, 1.8 mM CaCl_2_, 1 mM MgCl_2_, 10 mM D-glucose, 10 mM HEPES, pH 7.4 (adjusted by NaOH)]. Images were acquired by Leica SP8 confocal microscope with oil-immersion 63 × objective in a temperature-controlled chamber (37 °C). XYT scanning mode was adopted, and images in a size of 512 × 512 pixels at a scan speed of 400 Hz were captured every minute for a total of 22 minutes. TRPA1 blocker Chembridge-5861528 (CHEM, 10 μM, Alomone) or TRPA1 activator Allyl isothiocyanate (AITC, 150 μM, Sigma-Aldrich) or 0.1% DMSO (solvent control) was added into the confocal dish immediately before image acquisition. All cells were imaged with less than 1% laser power. Cells were excluded from analysis if they displayed signs of phototoxicity, such as shrinking.

For fixed-cell imaging, mESC-CMs were treated with TRPA1 blocker (CHEM, 10 μM) or activator (AITC, 150 μM) or 0.1% DMSO for different periods (30 minutes, 1 hour, 2 hours, and 4 hours) at 37 ℃ in 5% CO_2_ incubator. After drug incubation, the cells were stained with 100 nM MitoTracker Red in regular differentiation media for 30 minutes at 37 ℃ in a 5% CO_2_ incubator. Then immunocytochemistry was performed as described in Section 2.6. For TRPA1 or PGC-1α overexpression and TRPA1 knockdown, the cells were stained with 100 nM MitoTracker Red before fixation. α-actinin was used to identify mESC-CMs. Images were acquired by Leica SP8 confocal microscope with oil-immersion 63 × objective. XYZ scanning mode was adopted and images in size of 2048 × 2048 pixels at a scan speed of 100 Hz were captured.

Images of mitochondria obtained as described above were used to analyze mitochondrial dynamics and biogenesis. The images were segmented using the pixel classification feature of the interactive learning and segmentation toolkit software (Ilastik 1.3.0, European Molecular Biology Laboratory) and analyzed by Fiji [4]. Measures of mitochondrial area, number, and fractional occupancy were generated using the 'analyze particles' function in Fiji. Measures of mitochondrial length, junctions (voxels with three or more neighbors), and branches (slab segments connecting endpoints to either junctions or other endpoints) were determined using the 'skeletonize' and 'analyze skeleton' plugins in Fiji.

**Mitochondrial ROS measurements**

mESC-CMs were treated with the TRPA1 blocker (CHEM, 10 μM) or TRPA1 activator (AITC, 150 μM) or 0.1% DMSO for four hours at 37 ℃ in a 5% CO_2_ incubator. Mitochondrial ROS level was measured using CellROX Green (Thermo Fisher Scientific) which is weakly fluorescent while in a reduced state and exhibits bright green photostable fluorescence upon oxidation by ROS and subsequent binding to DNA, with absorption/emission maxima of ~ 485/520 nm. After four hours of treatment with different drugs, 2.5 μM CellROX Green was loaded into mESC-CMs in regular differentiation media for 30 minutes at 37 ℃ in a 5% CO_2_ incubator. After staining, the cells were washed with Tyrode's solution and bathed in 1 mL Tyrode's solution. Images were acquired by Leica SP8 confocal microscope with oil-immersion 63 × objective in a temperature-controlled chamber (37 °C). XYZ scanning mode was adopted and images in a size of 2048 × 2048 pixels at a scan speed of 100 Hz were captured. The intensity of CellROX Green was analyzed by Fiji.

**Mitochondrial membrane potential (ΔΨm) measurements**

mESC-CMs were treated with the TRPA1 blocker (CHEM, 10 μM) or TRPA1 activator (AITC, 150 μM) or 0.1% DMSO for four hours at 37 ℃ in a 5% CO_2_ incubator. Mitochondrial membrane potential (ΔΨm) was measured using the positively charged dye tetramethylrhodamine methyl ester (TMRE, Thermo Fisher Scientific). After four hours of treatment with different drugs, 20 nM TMRM was loaded into mESC-CMs in regular differentiation media for 30 minutes at 37 ℃ in a 5% CO_2_ incubator. After staining, the cells were washed with Tyrode's solution and bathed in 1 mL Tyrode's solution. Images were acquired by Leica SP8 confocal microscope with oil-immersion 63 × objective in a temperature-controlled chamber (37 °C). XYZ scanning mode was adopted and images in a size of 2048 × 2048 pixels at a scan speed of 100 Hz were captured. Then the intensity of TMRE was analyzed by Fiji.

**Measurements of mitochondrial respiration**

Mitochondrial oxidative characteristics of the mESC-CMs were tested by examining cellular oxygen consumption rates (OCR) using XF Long Chain Fatty Acid Oxidation Stress Test Kit (Agilent Technologies) with a Seahorse XF-96 Analyzer (Agilent Technologies). Isolated mESC-CMs were infected with adenovirus on day (7 + 5). For TRPA1 overexpression, experiments were performed on day (7 + 7). For TRPA1 knockdown, experiments were performed on day (7 + 9). One day prior to the Seahorse assay, mESC-CMs were trypsinized and plated in the Seahorse XF Microplate (Agilent Technologies) at a density of 5000 cells per well in the regular differentiation medium. On the day of the Seahorse assay, the growth medium was removed and the cells were washed once with the warmed assay medium (Agilent Technologies). 180 μL assay medium was added to each well and the plate was incubated at 37 °C in a non-CO_2_ incubator for 45 to 60 minutes prior to the assay. When performing the assay, baseline OCR was recorded first. Then 1.5 μM Oligomycin, which inhibits ATP synthase, was injected into the plate. It decreased electron flow through the ETC, resulting in a reduction in mitochondrial respiration or OCR. 1 μM FCCP, which is an uncoupling agent that collapses the proton gradient and disrupts the mitochondrial membrane potential, was the 2nd injection following oligomycin. The FCCP-stimulated OCR can be used to calculate spare respiratory capacity, defined as the difference between maximal respiration and basal respiration. Spare respiratory capacity is a measure of the ability of the cell to respond to increased energy demand or under stress. The third injection is a mixture of 0.5 μM rotenone, a complex I inhibitor, and antimycin A, a complex III inhibitor. This combination shuts down mitochondrial respiration and enables the calculation of nonmitochondrial respiration driven by processes outside the mitochondria. After the assay was completed, the data was analyzed with Agilent Seahorse Analytics (https://seahorseanalytics.agilent.com).

**Confocal Ca^2+^ imaging**

Cytosol Ca^2+^ transient was measured by using Fluo-4 (Thermo Fisher Scientific). mESC-CMs were stained with 5 μM Fluo-4 at 37 °C for 15 minutes, followed by washing with Tyrode's solution. After loading Fluo-4, the cells seeded on 25 mm coverslip were placed in the perfusion chamber and perfused with the required solutions. Briefly, cells were perfused with Tyrode’s solution for 1-2 minutes, followed by Tyrode’s solution containing different drugs. Leica SP8 confocal microscope was used to obtain data. XYT scanning mode was adopted and images in a size of 16 × 16 pixels were captured at a scan speed of 1400 Hz for each beating mESC-CM. Cells were imaged with oil-immersion 63× objective with excitation at 488 nm and emission at 520 nm. The pinhole was set to the largest and the laser power was set to 0.1%. Obtained data were analyzed with Fiji.

**Electrophysiology**

Membrane potential was measured with the ruptured whole-cell patch clamp. Microelectrodes were pulled from glass capillary (World Precision Instruments, Sarasota, FL, USA) by a pipette puller (Model P-97, Sutter Instrument, Novato, CA, USA) and polished with micro forge (NARISHIGE, Japan). The microelectrodes used in experiments were typically 3-6 MΩ after filling with the internal solution. Both microelectrodes and solutions were prepared freshly before experiments. Axopatch 200B amplifier (Molecular Devices, Sunnyvale, CA, USA) was used to amplify the signal detected by the microelectrode. pClamp 10.4 software (Molecular Devices) was used for recording signals.

For the action potential (AP) recordings, the cover glass seeded with mESC-CMs was placed into the temperature-controlled (33 °C) recording chamber and bathed in Tyrode's solution. The pipette solution contained: 50 mM KCl, 80 mM potassium aspartate, 1 mM MgCl_2_, 10 mM EGTA, 10 mM HEPES, and 3 mM Mg-ATP, adjusted pH to 7.4 with KOH. AP data were analyzed with Cardiac Action Potential Analysis Software (CAPA) Package Distributed by Science Consulting Cardiac Cellular Electrophysiology UG (Essen, Germany) [5].

**Western blotting**

Western blot was done as previously described [1-3]. 50 μg of protein lysate was separated on 7.5% or 10% SDS-polyacrylamide gels and electrophoretically transferred onto 0.45 μm PVDF membranes (Merck KGaA). The membranes were blocked with 5% (w:v) non-fat dry milk (Bio-Rad) in TBST for 1 hour at room temperature and incubated with primary antibodies at 4 °C overnight. On the next day, after washing with TBST three times, membranes were incubated with a secondary antibody in TBST for 1 hour at room temperature and developed using Clarity Western ECL Substrate (Bio-Rad) by Bio-Rad ChemiDoc MP System (Bio-Rad). Primary antibodies used in the study were as follows: anti-TRPA1 (1:1000, Alomone), anti-TRPA1 (1:1000, Novus Biologicals, CO, USA), MAPK Family Antibody Sampler Kit (1:1000, Cell Signaling Technology), Phospho-MAPK Family Antibody Sampler Kit (1:1000, Cell Signaling Technology), anti-MKP-1 (1:1000, Thermo Fisher Scientific), anti-PGC-1α (1:1000, Abcam), anti-β-actin (1:1000, Abcam), anti-β-tubulin (1:1000, Cell Signaling Technology). Secondary antibodies used were: HRP-conjugated goat anti-rabbit secondary antibody (1:3000, Dako), HRP-conjugated goat anti-mouse secondary antibody (1:3000, Dako).

**Quantitative Real-Time PCR (qRT-PCR) measurements**

Total RNA was extracted using Trizol reagent (Thermo Fisher Scientific). Genomic DNA was removed using TURBO DNA-free DNase Treatment and Removal Reagents (Thermo Fisher Scientific). SuperScript III Reverse Transcriptase (Thermo Fisher Scientific) was used to do reverse transcription. TB Green Premix Ex Taq (Tli RNaseH Plus) (TaKaRa, Kyoto, Japan) was used for intercalator-based real-time PCR with CFX96 Real-Time PCR Detection System (Bio-Rad). Relative quantification of target gene expression was performed using the 2^-ΔΔCt^ method. Target gene expression was normalized to the housekeeping gene and the relative gene expression of the target gene in different groups were normalized to that of the control group. Primers for qPCR were designed using Primer-Blast software by NCBI. Sequences of primers are shown in Table S1.

**Drugs and chemical reagents**

SB 203580, a specific inhibitor of p38 MAPK, was purchased from Tocris (Bristol, UK). All chemical reagents used to set up buffer solutions were purchased from Sigma-Aldrich.

**Statistical analysis**

Unpaired, two-tailed student's t-test was used for the statistical analysis using the GraphPad Prism 7 (GraphPad Software, Inc., La Jolla, CA, USA). Data were presented as mean ± SEM from at least 3 independent biological replicates. *P* < 0.05 was considered to indicate statistically significant differences.

**SUPPLEMENTARY FIGURE LEGENDS**

**Supplementary Figure 1. Expression of TRPA1 in mESC-CMs.** **A,** Total protein lysates from mESCs, EBs on day (7 + 4), EBs on day (7 + 8), EBs on day (7 + 12), and adult mouse heart. β-actin was used as a housekeeping gene for western blot. **B,** mESC-CMs at day (7 + 11) stained with anti-TRPA1 (green) and anti-α-actinin (red). Upper panel showed the staining of TRPA1 in α-actinin-positive cells; lower panel showed that peptide preincubation diminished the TRPA1 signal in mESC-CMs, verifying the specificity of anti-TRPA1 antibody. DAPI (blue) was used to stain the nuclei. Scale bar = 25 μm. **C,** Levels of mRNA transcripts encoding TRPA1 in control or TRPA1 OE mESC-CMs as measured by qPCR. Data were from 7 independent batches of differentiation. **D,** Representative western blots and bar graph showing the expression of TRPA1 in OE control or TRPA1 OE mESC-CMs. Data were from 3 independent batches of differentiation. **E,** Levels of mRNA transcripts encoding TRPA1 in control or TRPA1 KD mESC-CMs. Data were from 7 independent batches of differentiation. **F,** Representative western blots and bar graph showing the expression of TRPA1 in control or TRPA1 KD mESC-CMs. Data were from 3 independent batches of differentiation. Results were expressed as mean ± SEM. *, P < 0.05; ***, P < 0.001.

**Supplementary Figure 2. TRPA1 activity affected the Ca^2+^ handling and electrophysiological properties** **of mESC-CMs. A-C,** Representative traces of CaTs in mESC-CMs before and after treatment with 0.1% DMSO (solvent control) or 10 μM CHEM (TRPA1 blocker) or 150 μM AITC (TRPA1 activator). **D-E,** Bar graphs showing the time-to-peak and the decay time of CaTs in mESC-CMs treated with DMSO or CHEM or AITC. n = 13-14 cells from 3 independent batches of differentiation. **F-H,** Representative spontaneous AP tracings before and after treatment with 0.1% DMSO or 10 μM CHEM or 150 μM AITC. **I-J,** Bar graphs showing the DDR, V_max_-upstroke, MDP, and amplitude of APs. n = 6-7 cells from 3 independent batches of differentiation. Data were presented as mean ± SEM. *, P < 0.05; **, P < 0.01; ***, P < 0.001.

**Supplementary Figure 3. TRPA1 activity affected the biogenesis and dynamics of mitochondria in mESC-CMs. A,** Confocal time series of MitoTracker Red-labelled mitochondria in mESC-CMs treated with 10 μM CHEM, 150 μM AITC, or 0.1% DMSO. Scale bar = 10 μm. **B,** Line graphs showing the morphological changes of CHEM- or AITC- or DMSO-treated mitochondria. Data were presented as mean ± SEM. n = 10-13 cells from 3 independent batches of differentiation. *, P < 0.05, **, P < 0.01 vs DMSO. **C,** Confocal microscopy images of MitoTracker Red-labelled mitochondria in mESC-CMs treated with 10 μM CHEM, 150 μM AITC, or 0.1% DMSO for 30 minutes, 1 hour, 2 hours, and 4 hours. Scale bar = 25 μm. **D,** Line graphs showing the morphological changes of CHEM-, AITC- or DMSO-treated mitochondria. Data were presented as mean ± SEM. n = 8-10 cells at each time point from 3 independent batches of differentiation. *, P < 0.05; **, P < 0.01, ***, P < 0.001 vs DMSO.

**Supplementary Figure 4. TRPA1 activity affected the function of mitochondria in mESC-CMs. A,** (Left panel) Representative confocal microscopy images of mESC-CMs stained with CellROX Green. Scale bar = 25 μm. (Right panel) Bar graph showing the mean gray value of CellROX Green in different groups. n = 18-19 cells from 3 independent batches of differentiation. **B,** (Left panel) Representative confocal microscopy images of mESC-CMs stained with TMRE. Scale bar = 25 μm. (Right panel) Bar graph showing the mean gray value of TMRE in different groups. n = 22-40 cells from 3 independent batches of differentiation.Data were presented as mean ± SEM. *, P < 0.05; **, P < 0.01; ***, P < 0.001.

**Supplementary Figure 5. PGC-1α improved the maturation of mESC-CMs. A,** Representative western blots and bar graph showing the expression of PGC-1α. **B,** Confocal microscopy images of α-actinin and mitochondria in mESC-CMs with OE control or PGC-1α OE. **C,** Bar graphs showing the percentage of cells with disorganized sarcomeres, cell size, and percentage of multi-nucleated cells in mESC-CMs with OE control or PGC-1α OE. n = 33-47 cells from 3 independent batches of differentiation. **D,** Bar graphs showing the mitochondrial size (average mitochondrial area and branch length), mitochondrial complexity (number of branches and junctions per mitochondrion), mitochondrial number, and fractional occupancy. n = 15-16 cells from 3 independent batches of differentiation. **E-F,** Representative spontaneous AP tracings of OE control or PGC-1α OE mESC-CMs. **G,** Bar graphs showing the DDR, V_max_-upstroke, MDP, and amplitude of APs. n = 10-13 cells from 3 independent batches of differentiation. Results were expressed as means ± SEM. *, P < 0.05; **, P < 0.01; ***, P < 0.001.

**Supplementary Figure 6.** Representative western blots and bar graphs showing the expression of EKR/JNK and phospho-ERK/JNK in NRVMs. Results were expressed as mean ± SEM from 3 independent isolation of NRVM. *, P < 0.05; **, P < 0.01; ***, P < 0.001.

**REFERENCES**

1. Liu X, Zhao R, Ding Q, Yao X, Tsang SY. TRPC7 regulates the electrophysiological functions of embryonic stem cell-derived cardiomyocytes. Stem Cell Res Ther 2021;12:262.

2. Zhao R, Liu X, Qi Z, Yao X, Tsang SY. TRPV1 channels regulate the automaticity of embryonic stem cell-derived cardiomyocytes through stimulating the Na(+) /Ca(2+) exchanger current. J Cell Physiol 2021;236:6806-6823.

3. Qi Z, Wang T, Chen X, Wong CK, Ding Q, Sauer H, et al. Extracellular and Intracellular Angiotensin II Regulate the Automaticity of Developing Cardiomyocytes via Different Signaling Pathways. Front Mol Biosci 2021;8:699827.

4. Moore AS, Wong YC, Simpson CL, Holzbaur EL. Dynamic actin cycling through mitochondrial subpopulations locally regulates the fission-fusion balance within mitochondrial networks. Nat Commun 2016;7:12886.

5. Thieleczek R, Chang-Liao, M.-L., Zimmernann, H.-W. and Wettwer, E. Automated Cardiac Action Potential Analysis (CAPA). Acta Physiologica 2016;216:218-219.

**Table S1**

| **Primer name** | **Sequence (5’-3')** |
| --- | --- |
| HPRT forward | TGACACTGGAAAAACAATGCA |
| HPRT reverse | GGTCCTTTTCACCAGCAAGCT |
| TRPA1 forward | ATGACGGATGCACACCTCTCCA |
| TRPA1 reverse | TGCGCCCATAACTGGCTGCAA |
| MYH6 forward | AACCAGAGTTTGAGTGACAGAATG |
| MYH6 reverse | ACTCCGTGCGGATGTCAA |
| MYL2 forward | CTCCAAAGAGGAGATCGACCAG |
| MYL2 reverse | TGTTTATTTGCGCACAGCCC |
| TNNT2 forward | GCCAAAGATGCTGAAGAAGGT |
| TNNT2 reverse | GCACCAAGTTGGGCATGAAG |
| Gja1 forward | CCAAGGAGTTCCACCACTTTG |
| Gja1 reverse | CCATGTCTGGGCACCTCTCT |
| SCN5A forward | TGTTCCCATCGCAGTGGCTGA |
| SCN5A reverse | ACTTGGGATTCCTGCTGTTTGC |
| KCNH2 forward | ACAGGCTGGAAACCCGGCTA |
| KCNH2 reverse | CGCCACGAACTGGGAAACCTGA |
| CASQ2 forward | ACATCGTGGCCTTTGCGGAGA |
| CASQ2 reverse | TCCCAGTAAGCAACAAGCAGTGGA |
| RYR2 forward | CACCAGTACGACACAGGCTT |
| RYR2 reverse | TGCGTTTGATGCTCTCATGC |
| ITPR3 forward | TGAGATCAGCGAGCCGGTGT |
| ITPR3 reverse | ACCTGCATTGGTCAGCTCGGT |
| SERCA2A forward | ACCTTTGCCGCTCATTTTCC |
| SERCA2A reverse | GCTGCACACACTCTTTACCG |
| CAV3 forward | TCAATGAGGACATTGTGAAGGTAGA |
| CAV3 reverse | CAGTGTAGACAACAGGCGGT |
| JPH2 forward | AGACCTACGCAGACGGAGGAAC |
| JPH2 reverse | AGAACGCACCACCACTGCCA |
| POLG forward | AAGCGGACCGAGGACTTGTG |
| POLG reverse | CTCATGGTTGGTGCAGGGAG |
| POLG2 forward | TGGGACGCAAGGGAAGCAAACT |
| POLG2 reverse | TCCGCCCATCTCGACCCTGTAT |
| CPT2 forward | TGACAGCCAGTTCAGGAAGACAG |
| CPT2 reverse | GCCTGAGATGTAGCTGGTGTGC |
| CPT1b forward | TGGGCACCTCTGGGAGTTTGT |
| CPT1b reverse | GGGTGGCAACGTGGTGTTTG |
| PGC1-α forward | CCATATTCCAGGTCAAGATCAAG |
| PGC1-α reverse | TCACATACAAGGGAGAATTTCG |
| PGC-1β forward | GCTGACGAGAAGTAAAAGAGGC |
| PGC-1β reverse | TCAGTGTATCTGGGCCAACG |
| NRF-2 forward | ACTCCAGAAGGAACAGGAGAAGGC |
| NRF-2 reverse | GGAATGTGGGCAACCTGGGAGT |
| PPAR-α forward | ATAATTTGCTGTGGAGATCGGCCT |
| PPAR-α reverse | AGGAGCTTTGGGAAGAGGAAGGTGT |
| ERRα forward | TGCTCAAGGAGGGTGTGCGT |
| ERRα reverse | TGCGACACCAGAGCGTTCACT |
| TFAM forward | GCAAAGGATGATTCGGCTCAGGGAA |
| TFAM reverse | TCGTCCAACTTCAGCCATCTGCT |
| TFB1M forward | CTTGAGGCTGACAGACAAGA |
| TFB1M reverse | AACCACCAGAAGCTCAGCAA |
| TFB2M forward | CCTTGGTCAGCAGGTGTTCCT |
| TFB2M reverse | CGCAGGAGTACAGATCGAACAGA |
| COX1 forward | TCACTACCAGTGCTAGCCGCA |
| COX1 reverse | AGAGAATTGGGTCCCCTCCTCCA |
| COX2 forward | ACCTGGTGAACTACGACTGCT |
| COX2 reverse | TTAGTCGGCCTGGGATGGCA |
| CD36 forward | ACTGTGGCTAAATGAGACTGGGAC |
| CD36 reverse | GCCATCTCTACCATGCCAAGGA |
| ACADVL forward | AGTCGCAGTGGTGAACTGGCA |
| ACADVL reverse | AGGGACCTTGAGGCTCTGGACA |
| ACAT2 forward | TGACAAGGAGATTGTGCCAGTGC |
| ACAT2 reverse | CGCCATCGTTCATTCCTGATGCG |
| MFN1 forward | GGCGTGATTTGGAAAACAGT |
| MFN1 reverse | TACTTGGTGGCTGCAGTTTG |
| MFN2 forward | TACTTGGTGGCTGCAGTTTG |
| MFN2 reverse | GCAGAACTTTGTCCCAGAGC |
| OPA1 forward | TGTGATTGAAAACATCTACCTTCCA |
| OPA1 reverse | TTTAAGCTTGATATCCACTGTGGTGT |
| DRP1 forward | GGTGGTCAGGAACCAACAAC |
| DRP1 reverse | GGTGGTCAGGAACCAACAAC |
| FIS1 forward | ACTACCGGCTCAAGGAATATGAA |
| FIS1 reverse | ACAGCCAGTCCAATGAGTCC |
| NDUFS7 forward | GCGCTCCGAAAGGTGTACGA |
| NDUFS7 reverse | TGCGGTCACAGCCACGAACA |
| ND1 forward | TTCTGCCAGCCTGACCCATAGC |
| ND1 reverse | ATGGGCCGGCTGCGTATTCTA |

**Table S1 Sequences of primers for qPCR.**
