# Supplementary material for: TRPA1 promotes the maturation of embryonic stem cell-derived cardiomyocytes by regulating mitochondrial biogenesis and dynamics
Source: Stem Cell Res Ther. 2023 Jun 7;14:158. doi: 10.1186/s13287-023-03388-3 (PMC10249273; doi:10.1186/s13287-023-03388-3)

**SUPPLEMENTARY FIGURE 1**


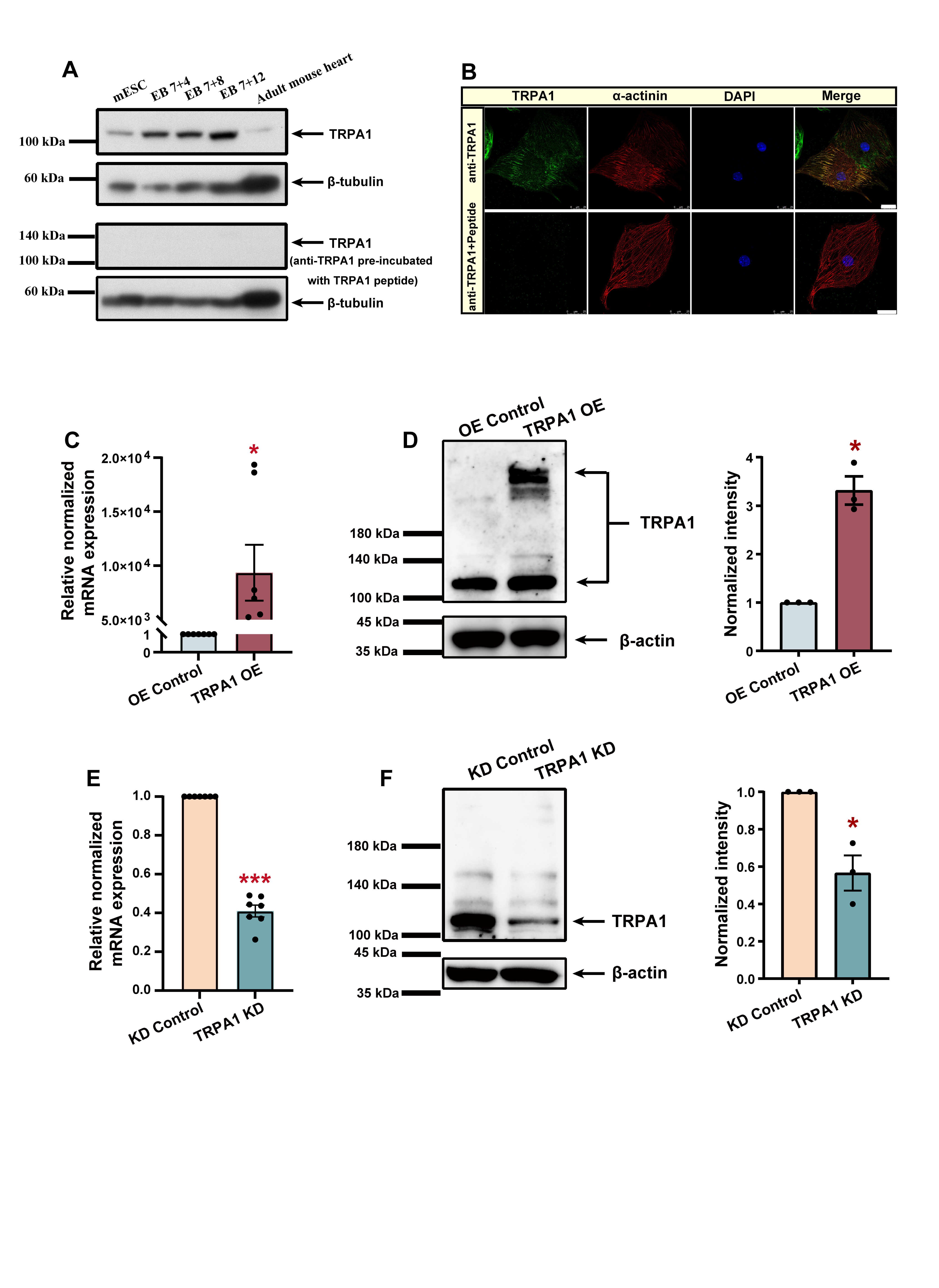


**SUPPLEMENTARY FIGURE 2**


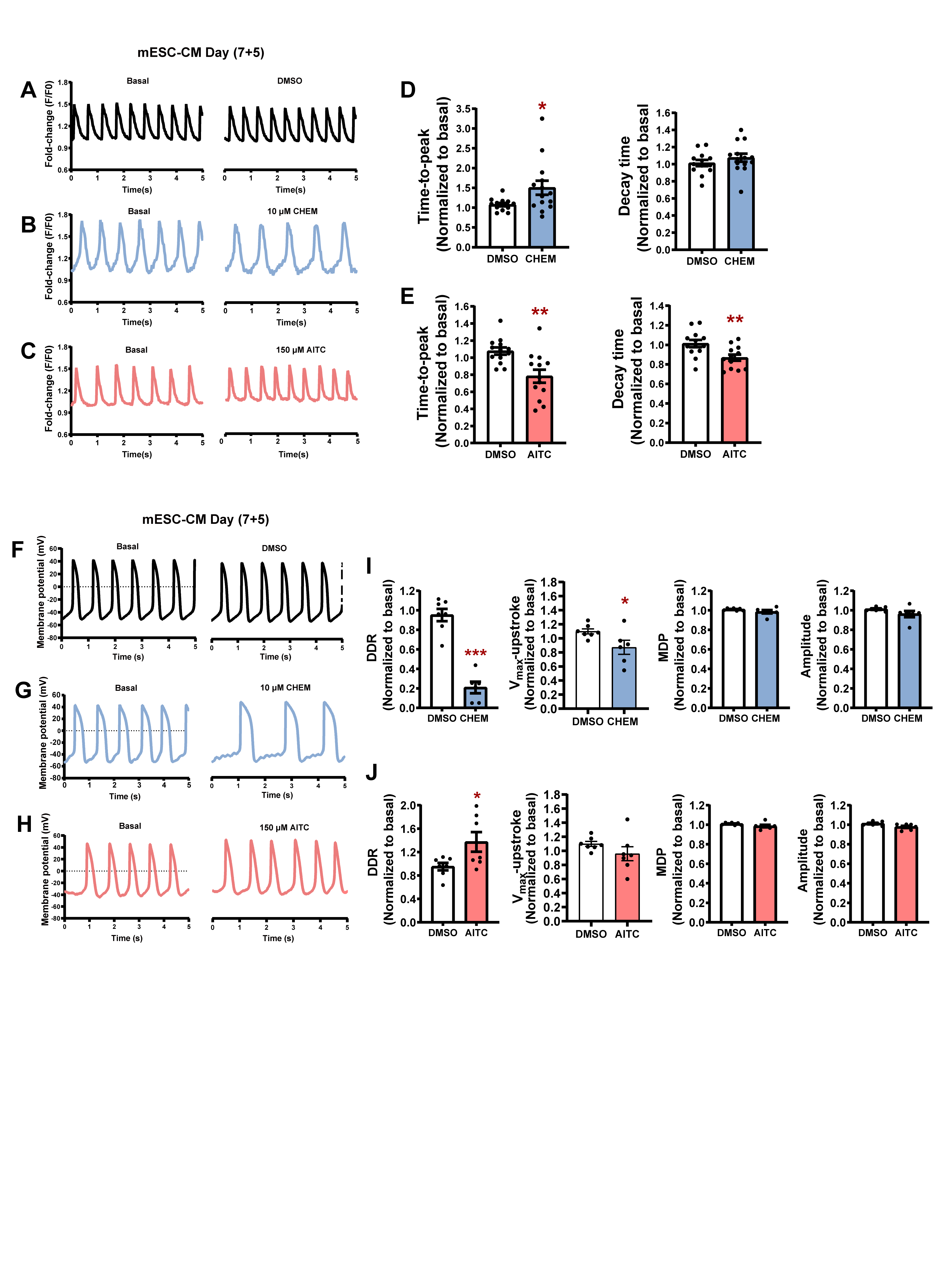


**SUPPLEMENTARY FIGURE 3**


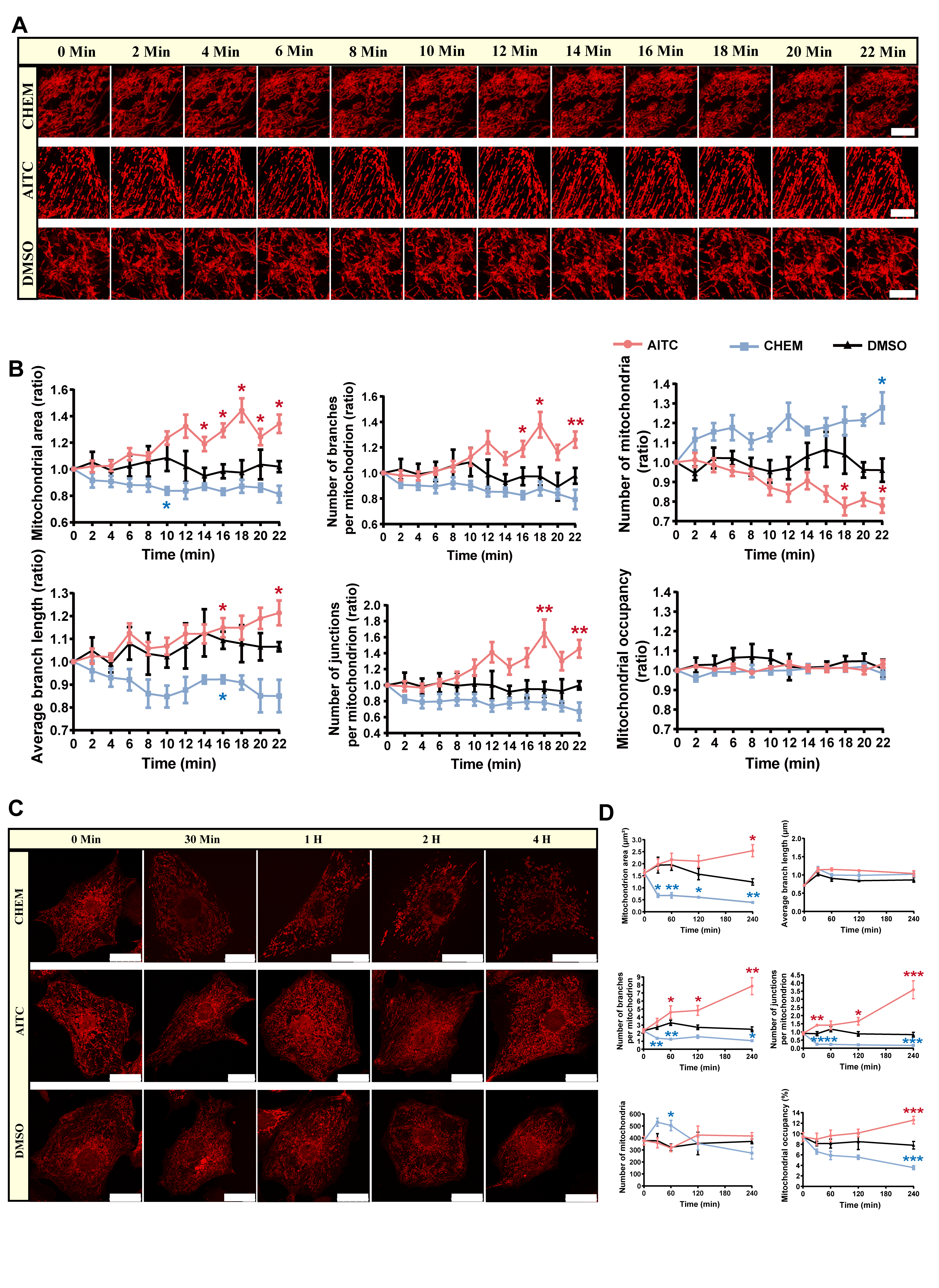


**SUPPLEMENTARY FIGURE 4**


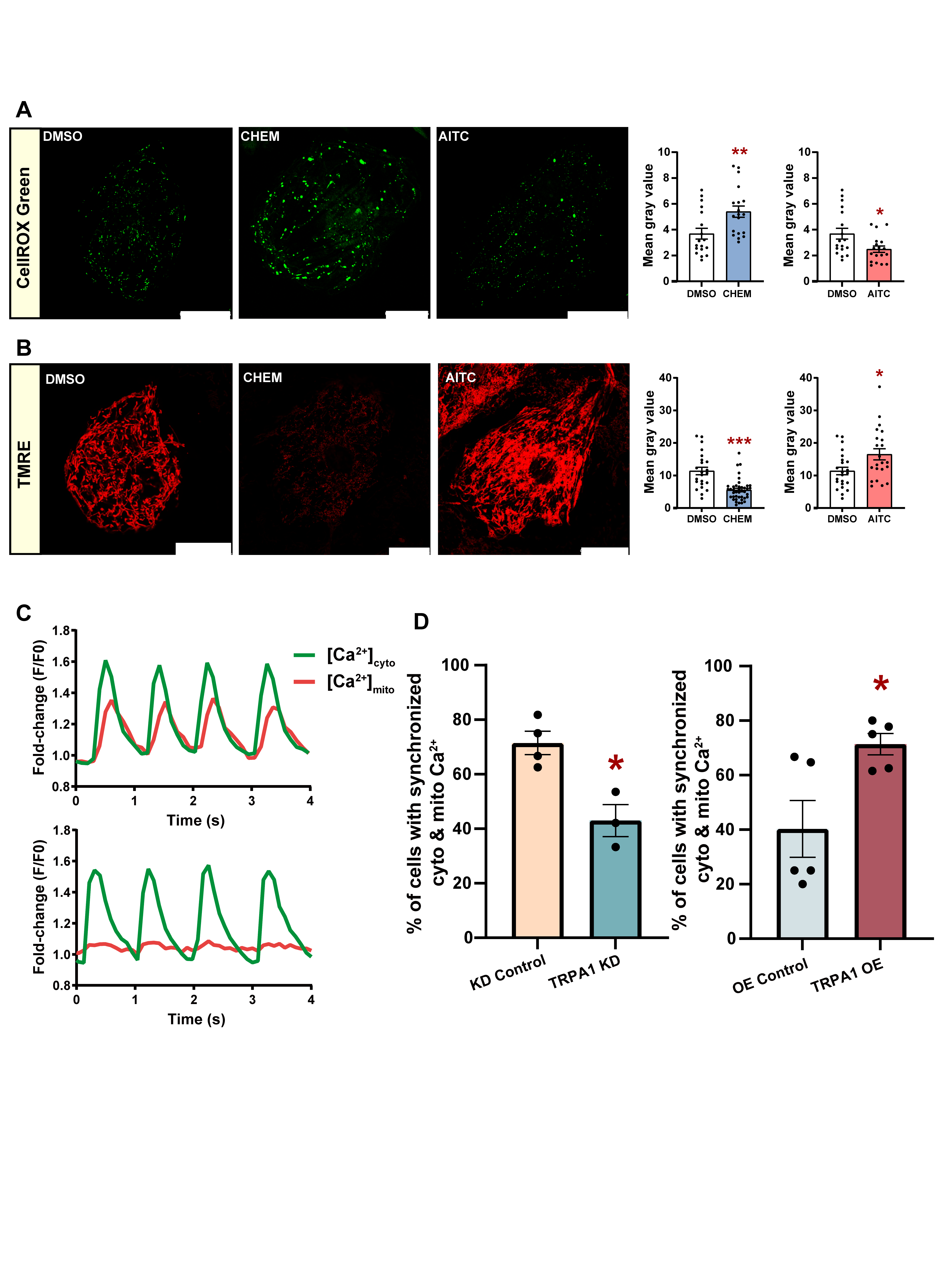


**SUPPLEMENTARY FIGURE 5**


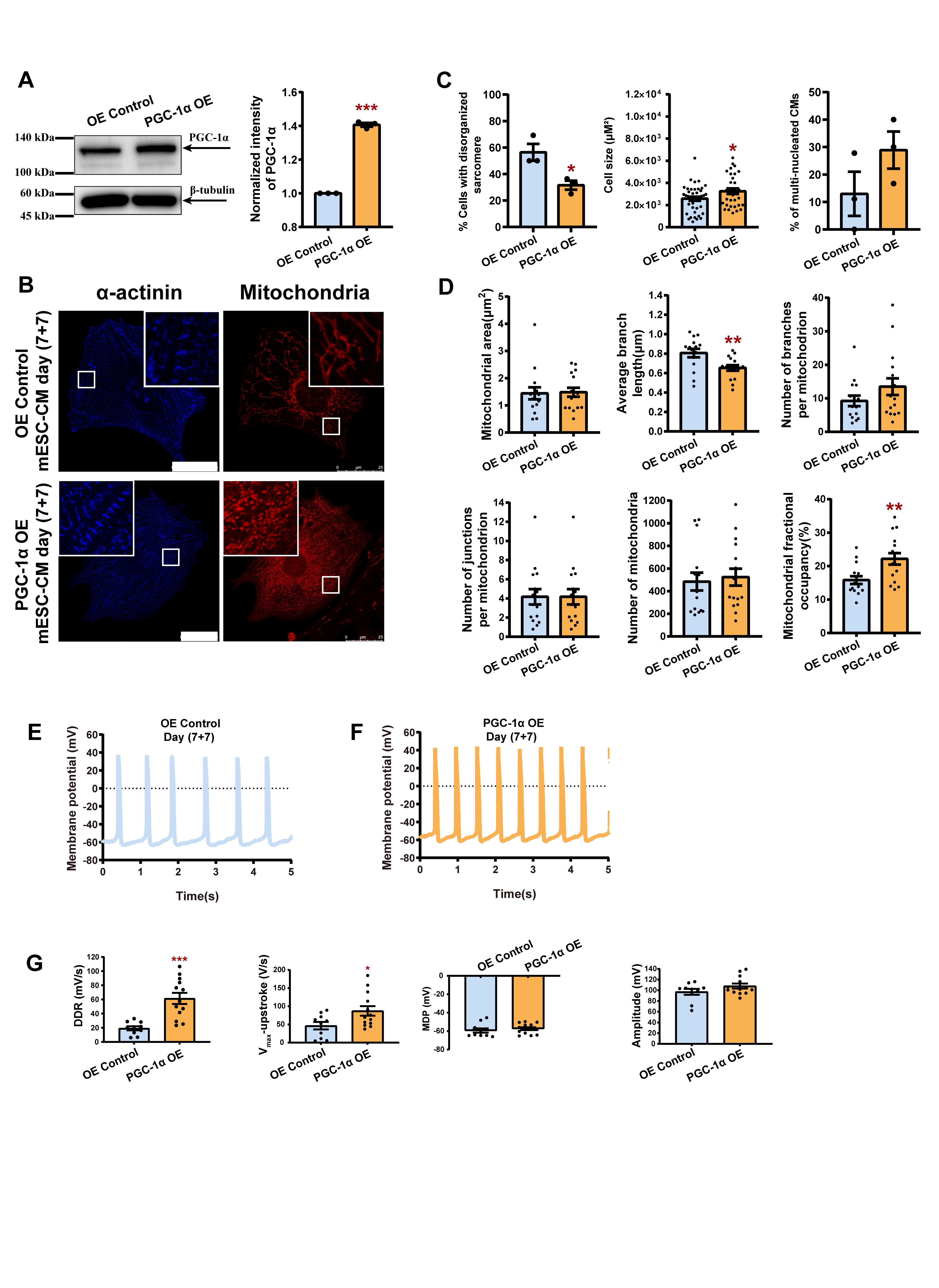


**SUPPLEMENTARY FIGURE 6**


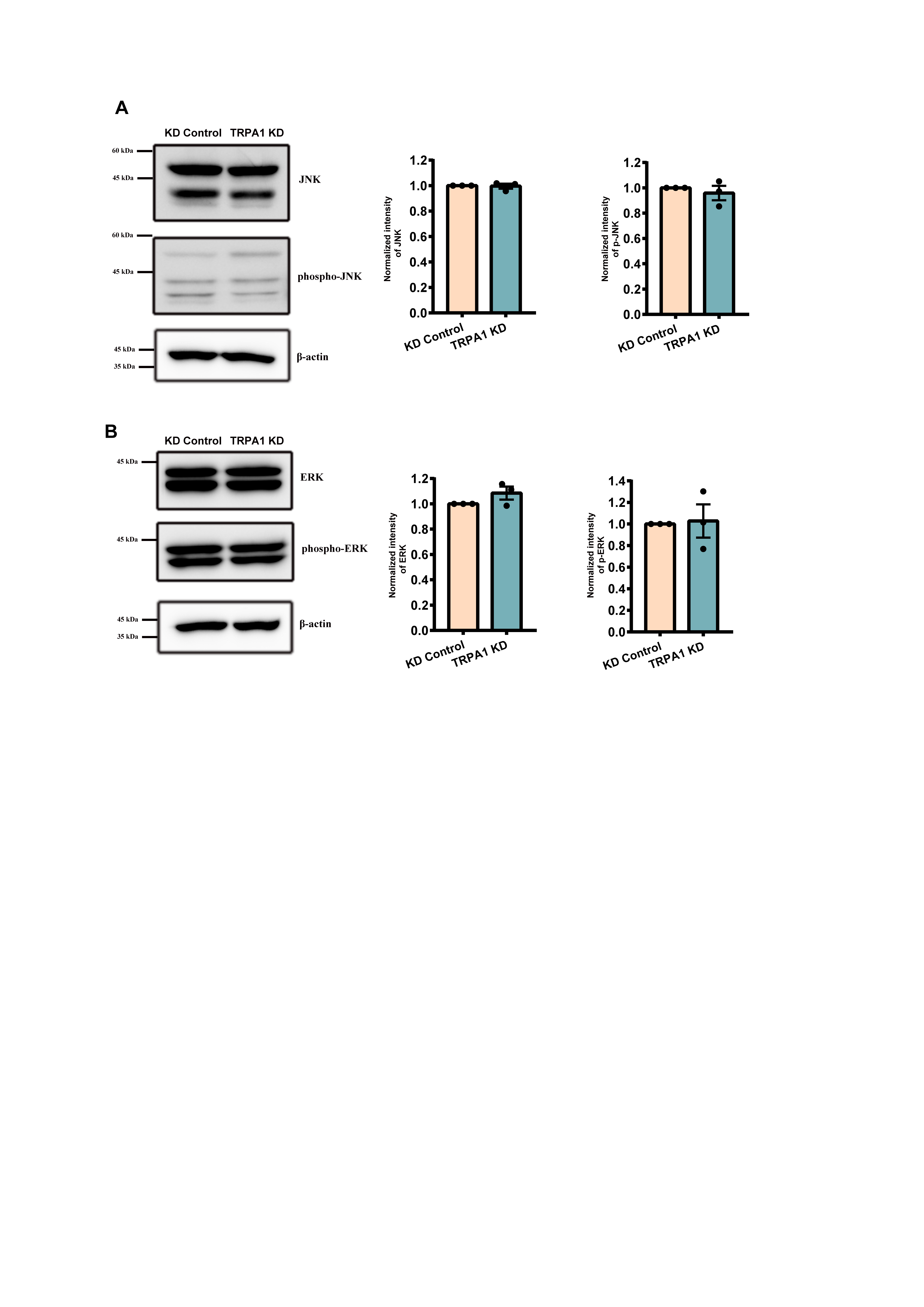

Supplement: Supplementary file 2 — Additional file 2. Supplementary Figures 1–6. [file 13287_2023_3388_MOESM2_ESM.docx]
