# Supplementary material for: TRPA1 promotes the maturation of embryonic stem cell-derived cardiomyocytes by regulating mitochondrial biogenesis and dynamics
Source: Stem Cell Res Ther. 2023 Jun 7;14:158. doi: 10.1186/s13287-023-03388-3 (PMC10249273; doi:10.1186/s13287-023-03388-3)
Supplement: Supplementary file 3 — Additional file 3. Supplementary Figure 7. [file 13287_2023_3388_MOESM3_ESM.docx]

**
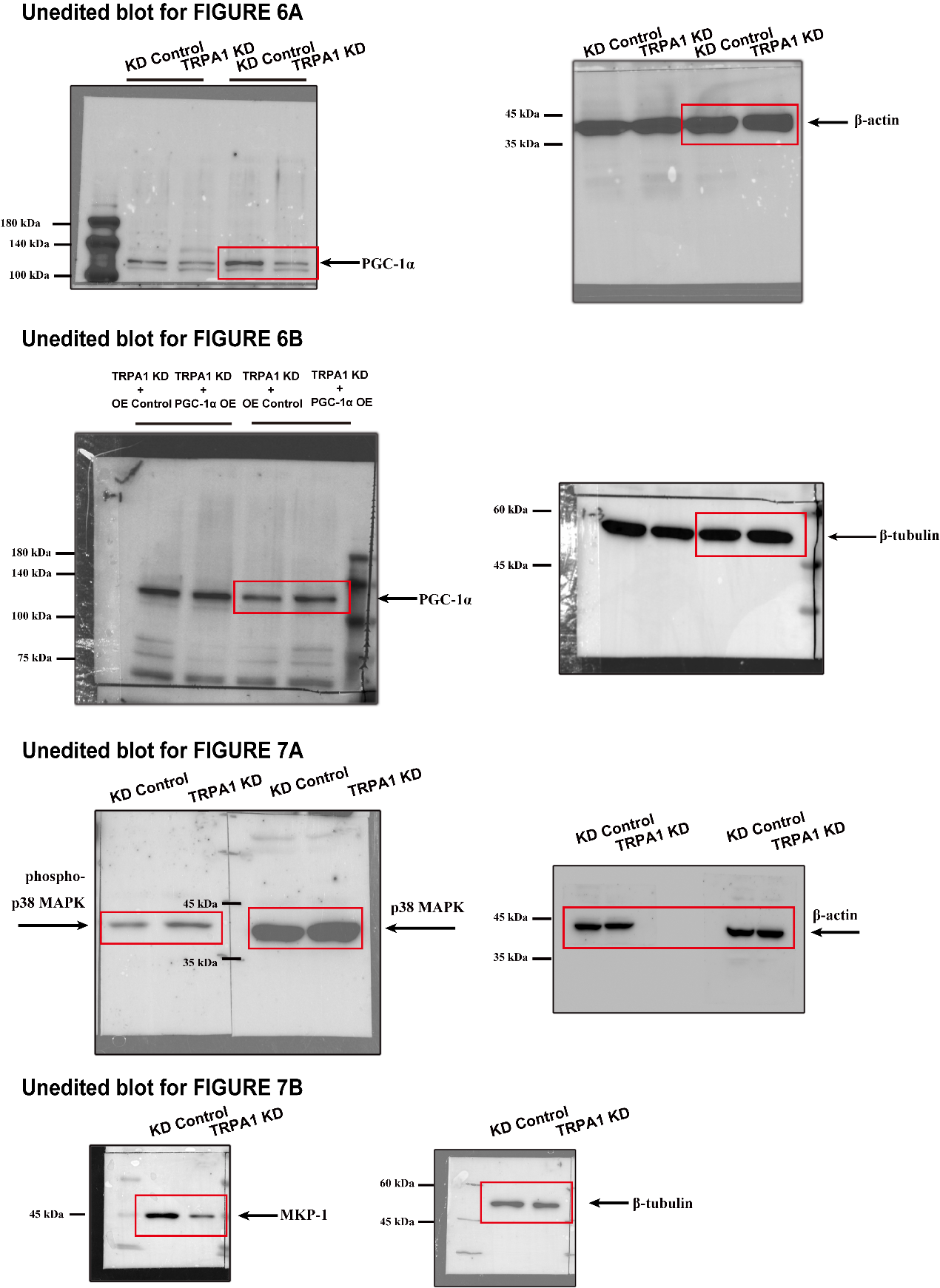
**

**
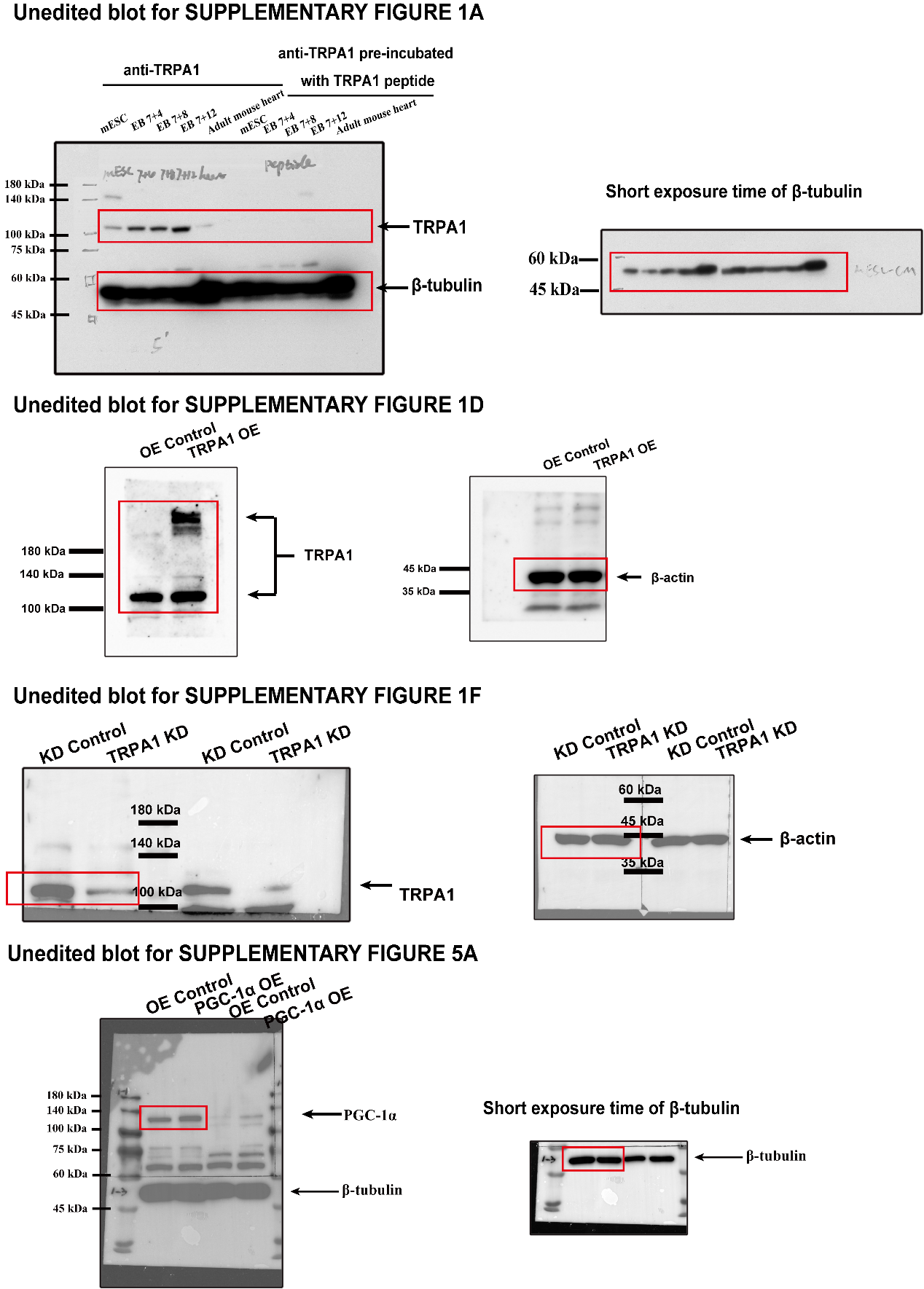
**

**
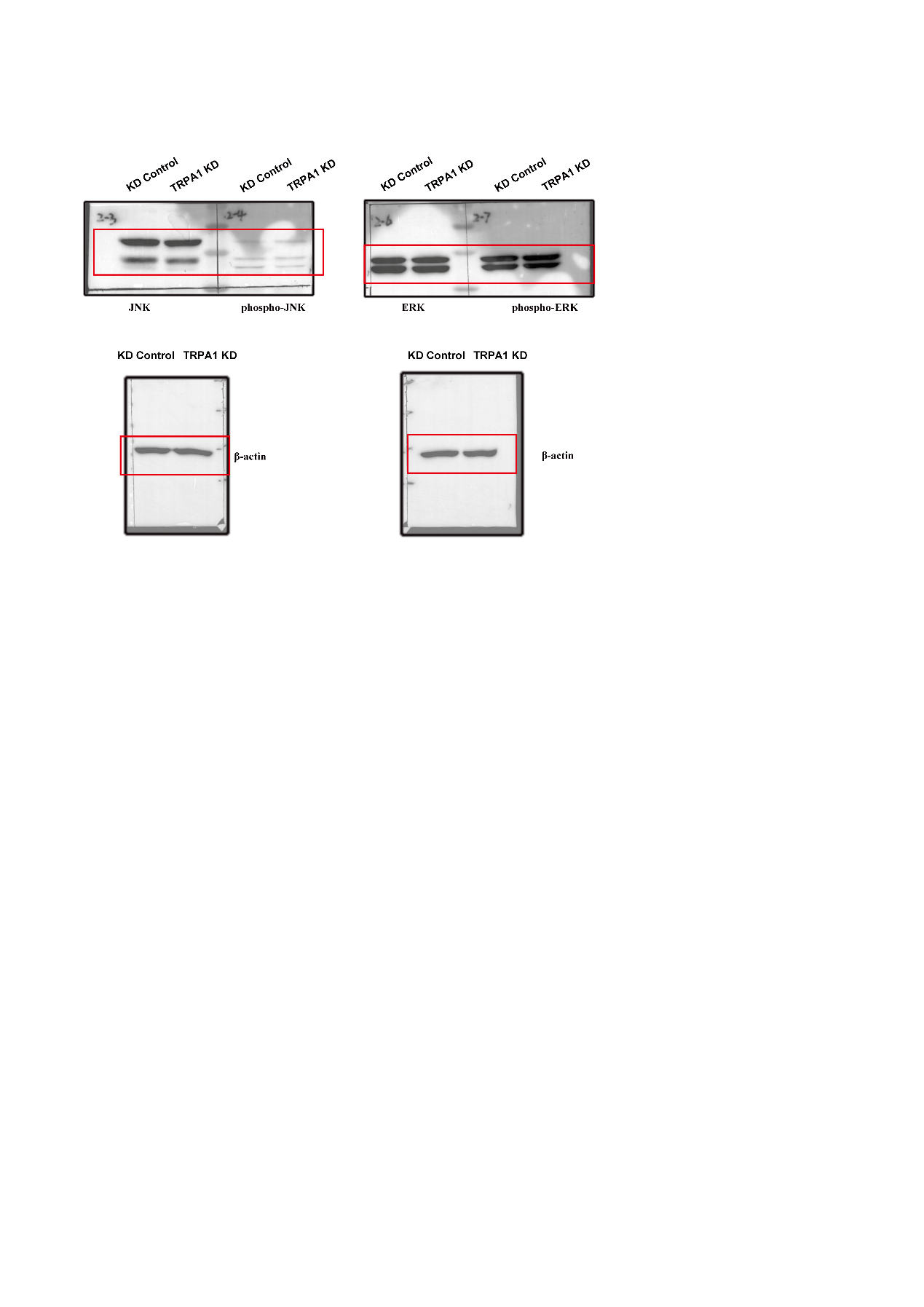
Unedited blot for SUPPLEMENTARY FIGURE 6**

**SUPPLEMENTARY FIGURE 7 LEGENDS**

To better show the edge of the membrane, we showed here the composite of chemiluminescence photo and colorimetric photo detected by Chemidoc. The bands in the red box indicate those shown in main and supplementary figures.

**Unedited blot for FIGURE 6A.** Unedited western blots showing the expression of PGC-1α in KD control and TRPA1 KD mESC-CMs.

**Unedited blot for FIGURE 6B.** Unedited western blots showing the expression of PGC-1α in TRPA1 KD mESC-CMs with or without PGC-1α overexpression.

**Unedited blot for FIGURE 7A.** Unedited western blots showing the expression of p38 MAPK and phospho-p38 MAPK in NRVMs

**Unedited blot for FIGURE 7B.** Unedited western blots showing the expression of MKP-1 in NRVMs.

**Unedited blot for SUPPLEMENTARY FIGURE 1A.** Unedited western blots showing the expression of TRPA1 in mESCs, EBs on day (7 + 4), EBs on day (7 + 8), EBs on day (7 + 12), and adult mouse heart. β-actin was used as a housekeeping gene for western blot.

**Unedited blot for SUPPLEMENTARY FIGURE 1D.** Unedited western blots showing the expression of TRPA1 in OE control or TRPA1 OE mESC-CMs.

**Unedited blot for SUPPLEMENTARY FIGURE 1F.** Unedited western blots showing the expression of TRPA1 in control or TRPA1 KD mESC-CMs.

**Unedited blot for SUPPLEMENTARY FIGURE 5A.** Unedited western blots showing the expression of PGC-1α OE control or PGC-1α OE mESC-CMs.

**Unedited blot for SUPPLEMENTARY FIGURE 6.** Unedited western blots showing the expression of ERK/JNK and phospho-ERK/JNK.
